# Non-Metal Primary Crowns in Telescopic and Double-Crown Systems Involving Natural Teeth: A Scoping Review

**DOI:** 10.3390/dj14100687

**Published:** 2026-10-21

**Authors:** Andreea Angela Ștețiu, Andi Ciprian Drăguș, Mihai David, Mădălina Adriana Malița, Mihai Burlibașa, Andrei Vorovenci, Viorel Ștefan Perieanu, Mihaela Romanița Gligor, Elena-Cristina Marcov, Florentina Căminișteanu, Narcis Marcov, Liliana Burlibașa, Ruxandra Stănescu

**Affiliations:** 1 Department of Dental Medicine and Nursing, Faculty of Medicine, Lucian Blaga University of Sibiu, 550169 Sibiu, Romania; andreea.stetiu@ulbsibiu.ro (A.A.Ș.); romanita.gligor@ulbsibiu.ro (M.R.G.); 2 Department of Dental Technology, Faculty of Midwifery and Nursing, Carol Davila University of Medicine and Pharmacy, 050474 Bucharest, Romania; andi.dragus@drd.umfcd.ro (A.C.D.); mihai.david@umfcd.ro (M.D.); madalina.malita@umfcd.ro (M.A.M.); viorel.perieanu@umfcd.ro (V.Ș.P.); florentina.caministeanu@drd.umfcd.ro (F.C.); 3 Doctoral School, Carol Davila University of Medicine and Pharmacy, 020021 Bucharest, Romania; 4 Department of Operative Dentistry, Faculty of Dentistry, Carol Davila University of Medicine and Pharmacy, 010221 Bucharest, Romania; elena.marcov@umfcd.ro (E.-C.M.); narcis.marcov@umfcd.ro (N.M.); 5 Department of Genetics, Faculty of Biology, University of Bucharest, 060101 Bucharest, Romania; liliana.burlibasa@bio.unibuc.ro; 6 Discipline of Implant-Prosthetic Therapy, Faculty of Dentistry, Carol Davila University of Medicine and Pharmacy, 050474 Bucharest, Romania; ruxandra.stanescu@umfcd.ro

**Keywords:** telescopic denture, double crown, natural tooth abutment, zirconia primary crown, non-metal primary crown, retention force, removable prosthodontics, scoping review

## Abstract

**Background/Objectives:** This scoping review mapped clinical, in vitro, and in silico evidence on non-metal primary crowns in telescopic and double-crown systems involving natural teeth, including retention, wear, mechanical behavior, clinical outcomes, and auxiliary retention-control elements. **Methods:** PubMed/MEDLINE, Scopus, Dentistry & Oral Sciences Source through EBSCO, and Web of Science were searched for English-language reports published from 2015 onward. These limits and the exclusion of case reports were predefined in a written, unregistered protocol. Elicit provided supplementary citation discovery, and a targeted backward reference-list check was performed using the same eligibility criteria. Findings and extractable numerical retention outcomes were charted descriptively without formal critical appraisal or quantitative pooling. **Results:** Forty-eight reports were included, 40 in vitro, one in silico, and seven clinical reports, representing six identifiable cohorts. Two clinical reports arose from the same randomized cohort. The backward reference-list check identified nine additional eligible reports. Clinical evidence was concentrated on zirconia primary crowns with electroformed or electroplated gold secondaries, although two additional short-term trials evaluated zirconia with polymeric secondary structures. Retention varied with the complete material and design combination. Four laboratory reports examined auxiliary retention-control concepts; no clinical study isolated their effects. **Conclusions:** Clinical evidence remains very limited. Polymeric primary crowns and auxiliary retention-control elements were represented mainly by laboratory evidence. Retention should be interpreted as a system-level outcome. Mixed-support outcomes require support-specific interpretation, and this review does not establish comparative clinical superiority.

## 1. Introduction

Telescopic or double-crown removable prostheses combine fixed primary crowns on teeth or implants with secondary components incorporated into a removable prosthesis. Depending on the design, these systems can provide prosthesis guidance, retention, the splinting of abutments, and access for hygiene. Tooth-supported overdentures and double-crown prostheses remain relevant for selected partially edentulous patients, particularly when strategic abutments can be preserved and a removable design is indicated [1,2,3,4,5].

Non-metal ceramic and polymeric materials have been investigated as alternatives to metallic primary crowns. Zirconia, lithium disilicate, polyetheretherketone (PEEK), and polyetherketoneketone (PEKK) have been evaluated in different material pairings. Fiber-reinforced composites (FRCs) have also been tested as a secondary-crown material. Computer-aided design and computer-aided manufacturing (CAD/CAM) permit different combinations of crown geometry, material, and fabrication method [6,7,8].

Retention should not be interpreted as a property of the primary-crown material alone. Material pairing, taper angle, surface condition, fabrication accuracy, lubrication, aging, and the selected retention mechanism can each modify insertion and removal forces [6,7,8]. This system-level behavior is particularly important when evidence from one material pair or geometry is applied to another design.

Available clinical reports describe mainly zirconia primary crowns combined with electroformed or electroplated gold secondary copings [9]. Laboratory studies have evaluated a wider range of pairings and friction pins, intermediate elements, and occlusal stops [10,11,12,13]. These components have different mechanical purposes, and their clinical effects require separate consideration.

This scoping review aimed to map clinical, in vitro, and in silico evidence on non-metal primary crowns in telescopic and double-crown removable dental prosthetic systems. The review question was as follows: what evidence exists regarding retention, wear, mechanical performance, survival, complications, maintenance, and patient-reported outcomes in systems with or without auxiliary retention-control elements?

## 2. Materials and Methods

### 2.1. Review Design, Question, and Reporting

A scoping review was undertaken to map the extent, characteristics, and gaps of evidence on non-metal primary crowns in telescopic and double-crown removable dental prosthetic systems involving natural teeth. The population–concept–context framework comprised natural tooth abutments in tooth-supported or combined tooth–implant-supported systems (population); non-metal primary crowns, their material pairings, retention, wear, mechanical and clinical outcomes, and retention-control elements (concept); and clinical, in vitro, or in silico prosthodontic settings (context). Reporting was guided by PRISMA-ScR [14], and the selection diagram followed PRISMA 2020 conventions [15]. The completed PRISMA-ScR checklist is provided as Supplementary File S1. A written review protocol was prepared before screening and specified the review question, eligibility criteria, information sources, and planned evidence-charting approach. The protocol was not prospectively registered.

The review question was as follows: what evidence has been reported since 2015 on the material and design characteristics, retention and wear behavior, mechanical performance, survival, complications, maintenance, and patient-reported outcomes of telescopic or double-crown systems involving natural teeth and at least one non-metal primary crown?

### 2.2. Information Sources and Search Strategy

PubMed/MEDLINE, Scopus, Dentistry & Oral Sciences Source through EBSCO, and Web of Science were searched on 20 June 2026. The searches combined terms for telescopic or double-crown prostheses, non-metal materials, and removable dental prosthodontics. English-language publications from 1 January 2015 onward were eligible. The temporal boundary was prespecified to focus on the contemporary CAD/CAM-era evidence base and the diversification of ceramic and polymeric systems. It defines the review scope and does not imply that the earlier literature is irrelevant. The English-language restriction was also predefined to permit a consistent screening and data-charting framework across the review team and was retained to preserve the original scope. No study-design filter was applied to the database searches. Appendix A (Table A1) reproduces the original strings as actually run. Database-specific syntax and material terminology differed; broader descriptors and acronyms were used, but full terms such as polyetherketoneketone were not present in every database strategy.

Elicit Pro (Elicit, web-based platform) was used on 3 May 2026 as a supplementary semantic-search, citation-discovery, and relevance-triage route. The query was as follows: in telescopic or double-crown removable dental prostheses, how do non-metal primary copings, with or without auxiliary retention attachments, perform in terms of retention, survival, wear, complications, and maintenance? The first 1000 ranked records were reviewed for relevance using titles, abstracts, and available bibliographic information to identify potentially relevant citations. Elicit ranking was not equivalent to independent human screening, and Elicit did not make final eligibility decisions. This route added no unique included report beyond the structured searches. A targeted backward reference-list check examined the reference lists of the included reports and the principal relevant reviews already cited [4,5,6]. Potentially eligible citations were assessed using the unchanged date, language, system, material, and study-design criteria. Reports identified through this route were checked against the original included set and treated as a separate citation-searching pathway. Sixteen reports proceeded to full-text assessment, and none were present among the original 544 database records. No new systematic database search or forward citation tracking was performed.

### 2.3. Eligibility Criteria

Eligible reports were original clinical, in vitro, or in silico investigations of telescopic or double-crown systems containing at least one non-metal primary crown. Clinical studies had to include natural tooth support, alone or combined with implants. Laboratory or computational studies using standardized dies or implant analogs were retained as indirect crown-pair engineering evidence only when the experimental unit was the telescopic interface, at least one primary crown was an eligible non-metal material, and the outcome concerned crown-level retention, insertion/removal, wear, fit, material pairing, fracture, taper, fabrication, or related mechanical behavior. The die or implant analog had to serve essentially as a standardized mounting substrate, with implant-specific variables outside the central tested mechanism. Findings that fundamentally depended on implant connection, distribution, angulation, peri-implant bone, implant-specific support biomechanics, or implant-only prosthesis behavior were not considered evidence for natural tooth systems. Direct laboratory tooth-support models and combined-support models were distinguished from isolated crown-pair experiments. Eligible materials included zirconia and other ceramics, lithium disilicate, PEEK, PEKK, and related non-metal polymeric or composite materials. Metallic comparator groups were retained within otherwise eligible reports.

Reports were required to provide an eligible system characteristic or outcome, including material pairing, taper or fit concept, retention force or change, wear, fit, strain or stress, fracture or fatigue, prosthesis or abutment survival, biological or technical complications, maintenance, clinical function, or patient-reported outcomes. Mixed tooth–implant clinical reports were retained when natural-tooth-containing configurations were identifiable. Pooled outcomes that could not be separated by support type were mapped as indirect evidence and were not interpreted as tooth-only estimates.

Exclusion criteria were publication before 2015, a language other than English, case reports, reviews, conference abstracts without an eligible full report, exclusively implant-supported clinical systems or implant-specific laboratory outcomes, exclusively metallic primary crowns without an eligible non-metal comparator, absence of a telescopic or double-crown component, and absence of an eligible outcome. Exclusively clasp-retained removable prostheses and fixed prostheses were also excluded. Case reports were excluded under the prespecified framework because this review was intended to map material- and system-level performance, including retention, wear, mechanical behavior, survival, complications, maintenance, and patient-reported outcomes. Individual reports can demonstrate feasibility or clinical application but generally lack a meaningful denominator and cannot separate material, design, patient, and operator effects. Their exclusion was a scope decision, not a judgment that case reports are inherently invalid.

### 2.4. Record Management and Study Selection

The structured-search results were imported into EndNote X9 (Clarivate, Philadelphia, PA, USA) and deduplicated. Titles and abstracts and, subsequently, full texts were screened independently in duplicate by four reviewers working in pairs. Disagreements were resolved through discussion; unresolved decisions were adjudicated by two senior reviewers. Elicit rankings supported discovery and triage, while final eligibility decisions remained with the human review team. The targeted backward reference-list check was documented as a separate citation-searching route. Reports identified through this route were deduplicated against the included-report set before full-text assessment.

### 2.5. Data Charting and Evidence Synthesis

Data charting was performed independently in duplicate using piloted forms. Variables comprised author, publication year, country, design, sample size, prosthesis or specimen type, support model, primary and secondary materials, taper or fit concept, manufacturing method, retention-control element, aging or follow-up, retention and wear outcomes, mechanical outcomes, survival, complications, maintenance, and patient-reported outcomes. Reports from the same identifiable clinical cohort were linked and counted as separate reports without treating them as independent cohorts. The final published bibliographic year was used consistently. Funding and material-support statements were charted as reported in Supplementary Table S2; the absence of a statement was not interpreted as the absence of support. For reports providing retention or removal force outcomes, numerical outcomes and principal testing conditions were charted where extractable, with separate experimental-group rows, source locations, original units, and original summary measures. NR denotes information not reported or not numerically extractable. Values were not estimated from graphs. Directly reported changes were retained; calculated changes were explicitly identified and used only when the reported endpoints permitted an unambiguous comparison.

No quantitative pooling was planned or performed because the reports differed in design, material combinations, specimen units, taper definitions, loading, lubrication, cycle counts, and outcome measures. Evidence was summarized descriptively by evidence type, support model, material pairing, retention concept, aging or follow-up, and outcome. No formal risk-of-bias or methodological-quality assessment was performed because the purpose was to map the evidence rather than estimate comparative treatment effects. The amount and type of evidence were therefore distinguished from its methodological quality or certainty.

## 3. Results

### 3.1. Study Selection

**Figure 1:**
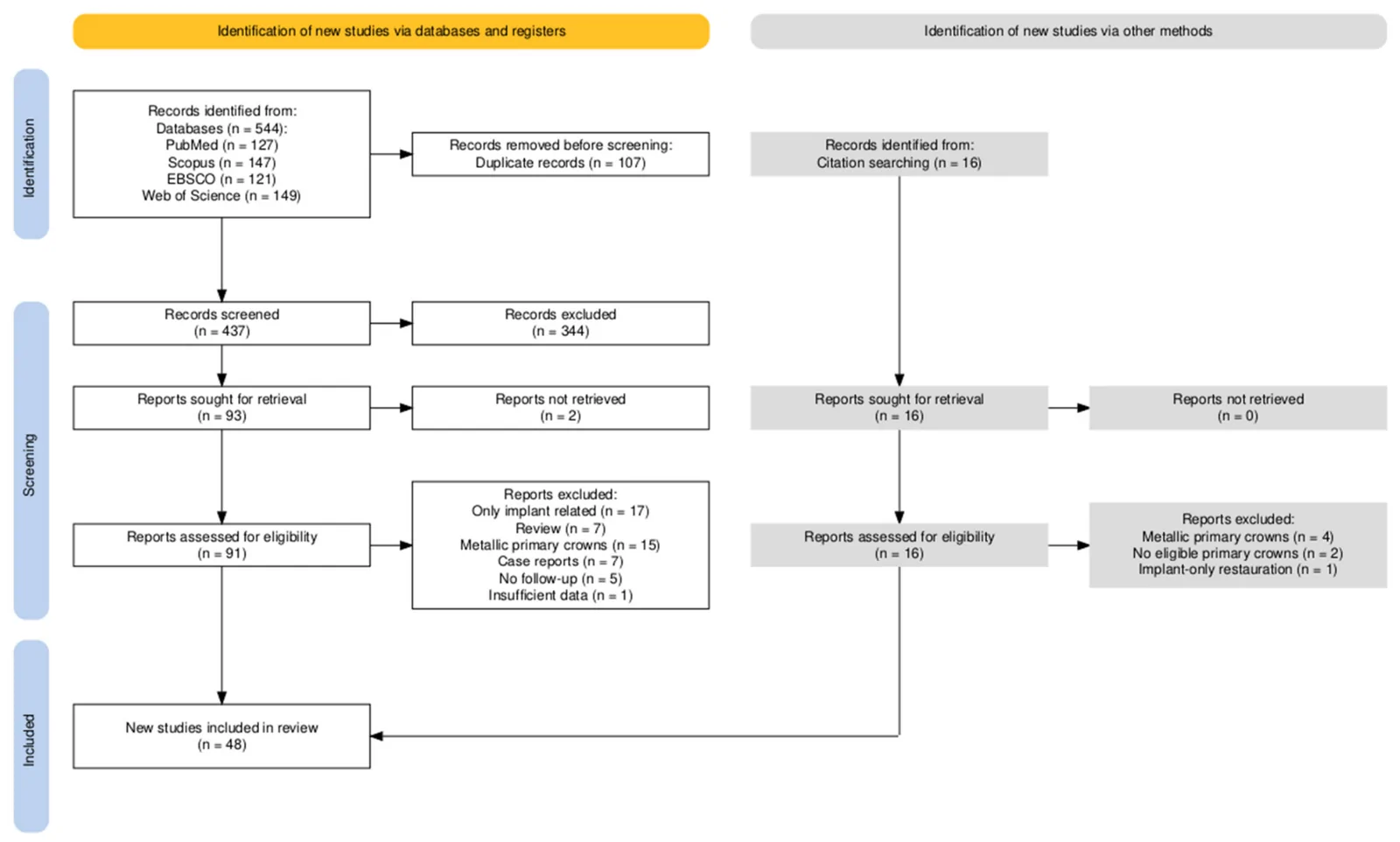
The PRISMA flow diagram. The selection of reports through the structured database searches and targeted backward reference-list checking. The citation-searching route was treated separately from the original 544-record database route. Sixteen reports were assessed at full text, nine were included, and seven were excluded. Elicit added no unique included report beyond the structured searches.

The structured database search identified 544 records: 127 from PubMed/MEDLINE, 147 from Scopus, 121 from Dentistry & Oral Sciences Source through EBSCO, and 149 from Web of Science. After the removal of 107 duplicates, 437 records were screened by title and abstract; 344 were excluded, and 93 reports were sought. Two reports could not be retrieved despite additional attempts to obtain the full texts, including author-contact attempts, leaving 91 reports available for full-text eligibility assessment. Fifty-two reports were excluded: 17 were exclusively implant-related, seven were reviews, 15 evaluated only metallic primary crowns, seven were case reports, five lacked eligible outcome or usable test/follow-up data, and one had an ineligible population or specimen. The structured database route therefore yielded 39 reports. Elicit added no unique included report beyond that route. The backward reference-list check identified nine additional eligible reports. The evidence map contains 48 reports: 40 in vitro, one in silico, and seven clinical reports, representing six identifiable cohorts (Figure 1; Table 1).

The separate backward reference-list route yielded 16 reports for full-text assessment that were not present among the original 544 database records. Nine were included, and seven were excluded: four evaluated only metallic primary crowns, two did not identify an eligible non-metal primary material, and one evaluated implant-only prosthesis behavior.

### 3.2. Characteristics of Included Studies

The included reports were published from 2016 to 2025. The seven clinical reports represented six identifiable cohorts: the two reports from the tooth-supported randomized trial of zirconia versus CoCr primary crowns with electroplated gold secondaries [16,17]; the mixed-support lithium-disilicate trial [18]; two retrospective ceramic or zirconia/gold datasets [9,19]; and two short-term trials of zirconia primary crowns with polymeric secondary structures [20,21]. The Brandt reports were retained as separate identifiable datasets because participant-level overlap could not be established. Table 2 presents clinical evidence only. Supplementary Table S1 contains the complete report-level evidence map.

Most laboratory reports evaluated retention, wear, taper, material pairing, fracture, or stress/strain. Tested primary materials included zirconia, ceria-stabilized zirconia/alumina (Ce-TZP/Al_2_O_3_), lithium disilicate, PEEK, PEKK, Bio-HPP, and graphene-reinforced acrylic, with metallic within-report comparators. FRC was tested as a secondary material. Awad et al. used a combined natural tooth/implant model [22]. Other reports used tooth-supported prosthesis models or standardized crown-pair tests, including implant-mounted pairs in which the mounting substrate was not the central experimental variable. These latter findings were mapped as indirect crown-pair engineering evidence.

### 3.3. Primary Coping and Secondary Structure Materials

Zirconia was the non-metal primary material represented the most often in the clinical reports. It was paired with electroformed or electroplated gold in four reports from three identifiable cohorts [9,16,17,19]. Two additional clinical cohorts used zirconia with PEEK, PEKK, or Bio-HPP secondary structures [20,21]. Laboratory reports examined zirconia with gold, zirconia, CoCr, PEEK, PEKK, FRC, and other secondary materials [7,10,13,23,24,25].

PEEK and PEKK primary crowns were represented by laboratory evidence. Reports also examined these polymers as secondary crowns on zirconia and other primaries. Some configurations showed stable or increased retention after cycling, whereas others showed retention loss [26,27,28,29,30,31,32,33,34]. The two additional clinical trials concerned polymeric secondary structures, not polymeric primary crowns [20,21].

Lithium-disilicate primary crowns were evaluated clinically by Zahn et al. [18]. That report described limited long-term primary-crown stability and more unfavorable outcomes for Vectris secondary structures than for electroplated gold within its mixed-support sample. Linek et al. also tested lithium-disilicate primaries with electroformed gold in vitro; data from that report give a mean pull-off force of 3.4 ± 1.1 N initially and 2.9 ± 0.8 N after 10,000 cycles [35]. These laboratory findings do not resolve the clinical limitations of the material.

### 3.4. Retention Behavior and Taper Angle

Retention was the most frequently charted laboratory outcome. Supplementary Table S3 presents quantitative outcomes and principal testing conditions for 33 laboratory reports that evaluated retention or removal/loosening force. Separate rows preserve group-specific material pairs, geometry, sample units, original summary statistics, aging conditions, and methodological qualifications. Some reports provided only graphical endpoints or selected numerical summaries, for which NR is used. The values were not pooled. Material pairing, taper, fit, surface condition, manufacturing method, lubrication, seating force, and cycling all affected reported retention [6,7,24,28,29].

Several reports described higher retention at smaller tapers, but the direction and magnitude depended on the tested system. Kawilarang et al. reported the highest initial values at 0° among the 0°, 1°, and 2° configurations [36]. Yoshikawa et al. reported less proportional retention loss at 2° than at 4° after 10,000 cycles [37]. Fischer et al. and Enaba also described geometry-dependent forces [8,38]. Two additional Nakagawa reports examined taper and fabrication variables in Ce-TZP/Al_2_O_3_ crown pairs [39,40]. Their numerical summaries use different experimental designs and should not be treated as directly comparable group estimates.

Taper did not act independently. Merk and Stock reported interactions with material pairing or secondary-crown fabrication [7,28,29]. Nakajima et al. described higher forces in water than under dry conditions for the same Ce-TZP/Al_2_O_3_ pairs [41]. Fujiki et al. described changes with surface treatment and seating load [42]. Other reports compared secondary materials, fabrication methods, lubrication, and tooth form [43,44,45,46]. These findings describe system behavior under specific test conditions and do not establish a universal material ranking.

### 3.5. Auxiliary Retention-Control Elements

Four laboratory reports evaluated retention-control concepts with non-metal primary crowns [11,12,13,47]. These reports investigated friction pins, intermediate elements, or occlusal stops, but the concepts and comparators were not interchangeable. Arnold et al. reported that the zirconia friction-pin group changed from 12.2 N to 12.4 N after aging, whereas a zirconia/electroplated gold group without pins lost 39% of retention [11]. Because the secondary material and fit concept also differed (CoCr clearance fit with gold friction pins versus electroplated gold), this was not a matched with-versus-without-pin comparison and does not isolate the pin effect.

Kochanek-Leśniewska et al. evaluated three-element systems with zirconia primary crowns and electroformed gold or milled polymethyl methacrylate (PMMA) intermediate elements [12]. The matched comparisons A versus B and C versus D did not establish a clear retention advantage of gold over PMMA. One control specimen lost retention at 16,800 cycles. A group-E run stopped at 10,000 cycles because the lower holder post fractured; this was a fixture failure. Absolute force endpoints were graphical and were not estimated for S3.

Occlusal stops limited excessive removal forces. Schwindling et al. reported mean maximum loosening forces of 12.1 N with zirconia stops and 12.8 N with composite stops [47]. Tasaka et al. used composite occlusal stops in zirconia/FRC conical crowns; the reported force means increased after aging [13]. These findings concern the regulation of maximum force rather than a general objective of increasing retention.

Kotthaus et al. did not test an auxiliary intra-prosthetic retention attachment. The grub screws in that experiment were used to mount the PEKK secondary crowns in the wear simulator; retention was generated by the crown pair itself [32]. Accordingly, the evidence map classifies this report under material pairing rather than auxiliary retention. Overall, the available data show that retention-control elements are design-specific: friction pins or intermediate layers may stabilize selected systems, whereas occlusal stops are intended to cap excessive forces. The evidence does not support a pooled estimate of an auxiliary-element effect.

### 3.6. Wear, Fracture, and Biomechanical Behavior

Wear findings varied with the material pair and test conditions. Pott et al. reported comparatively low secondary wear for zirconia/PEEK within their experiment and greater primary wear for PEEK/PEEK [48]. Milić-Lemić et al. compared early retention settling in zirconia/PEEK and zirconia/electroplated gold systems with zirconia tertiary structures [49]. Muwafi et al. reported greater wear for Bio-HPP than CoCr in digitally designed tooth-supported telescopic partial dentures [50]. These outcomes should not be interpreted as clinical durability estimates.

Arnold et al. described smaller retention losses in friction-pin groups than in several extended-contact designs [11]. Because material pairing and fit concept varied simultaneously, the independent contribution of the pin remains uncertain. The report documents the laboratory behavior of complete designs and cannot establish clinical survival or maintenance requirements.

Two laboratory reports examined fracture. Melitt et al. described higher fracture loads for zirconia than PEEK telescopic overdentures and different failure patterns [51]. Lümkemann et al. tested veneered high-performance polymer secondary crowns on zirconia primaries after 1.2 million chewing cycles and thermocycling [52]. Fracture loads are specific to specimen design and aging conditions and are not clinical survival probabilities.

The finite element report described changes in secondary-crown and cement-layer stress with the assigned primary-material elastic modulus [53]. Mohamed et al. compared strain patterns in zirconia- and PEKK-primary tooth-supported models [54]. A further Muwafi report described different abutment and ridge strain distributions after 240,000 loading cycles for CoCr and Bio-HPP telescopic partial dentures [55]. These model findings do not establish effects on clinical tooth survival or bone preservation.

### 3.7. Clinical Outcomes

Clinical evidence comprised seven reports from six identifiable cohorts. Available clinical evidence is concentrated on zirconia primary crowns combined with electroformed or electroplated gold secondary copings. Brandt et al. evaluated 126 prostheses: 19 tooth-only, 30 combined tooth–implant, and 77 implant-only restorations [9]. Overall five-year survival was 96.9%; five of six failures occurred in the tooth-only subgroup, or 26.3% of that subgroup, while the combined-support subgroup had 100% survival over a mean 44.48 months. The support-specific results are distinct from the pooled estimate. The tooth-supported Schwindling randomized cohort reported 96.4% prosthesis survival at three years in both material groups, with substantial aftercare [17].

In the same Schwindling cohort, Oral Health Impact Profile-49 scores improved after treatment, without a clear between-group difference at 12 months [16]. Brandt et al. reported high satisfaction over periods up to 16 years but did not separate outcomes by support type [19]. Zahn et al. included 77 natural teeth and 88 implants and reported long-term material-group differences; most prosthesis outcomes were pooled across support types [18]. Refai et al. studied 18 participants for one year with zirconia primary crowns and PEKK or PEEK secondary frameworks [20]. Reported baseline and one-year dislodging forces were 19.52 ± 0.88 and 25.02 ± 2.76 N for PEKK and 18.67 ± 0.53 and 20.10 ± 2.45 N for PEEK. The source’s separate change row is inconsistent with these endpoints and was not used for a change estimate. Elsarrif et al. studied 14 participants with zirconia/Bio-HPP systems for 12 months and reported different radiographic bone-height changes at abutment and residual-ridge sites between milled and pressed secondary frameworks [21]. Neither trial establishes long-term comparative survival.

No included clinical report isolated friction pins, intermediate retention elements, or occlusal stops with non-metal primary crowns. Clinical findings therefore concern complete crown and prosthesis systems rather than the independent effects of auxiliary retention-control elements.

### 3.8. Clinical and Report-Level Evidence Maps

Table 2 summarizes the seven clinical reports in six cohort-level rows and links the two Schwindling publications within a single row. The complete 48-report map is provided in Supplementary Table S1, and laboratory retention outcomes and testing conditions are provided in Supplementary Table S3.

### 3.9. Funding and Material Support of Included Reports

The source reports described a mixture of institutional or grant funding, material supply, manufacturing assistance, equipment provision, and open access publication funding. Several stated that they had received no study funding while also acknowledging other assistance. Supplementary Table S2 preserves these distinctions and records the absence of a located statement as not reported. Funding and support statements were charted descriptively and were not used as a surrogate risk-of-bias assessment.

## 4. Discussion

### 4.1. Principal Findings

Of the 48 included reports, 40 were in vitro, one was in silico, and only seven were clinical, representing six identifiable cohorts. Two Schwindling reports arose from the same randomized cohort [16,17]. Available clinical evidence is concentrated on zirconia primary crowns with electroformed or electroplated gold secondary copings [9,16,17,19]. Backward checking also identified two short-term clinical trials of zirconia with polymeric secondary structures [20,21]. The broader material range and most auxiliary retention-control concepts were represented by laboratory reports.

This numerical distribution describes the amount and type of evidence, not its certainty. The clinical samples were small or restricted to selected settings, follow-up varied, and some outcomes were pooled across support types. The additional polymer-secondary trials extend the clinical map but do not provide long-term survival comparisons [20,21]. The findings for zirconia cannot be generalized to all ceramics, as the lithium-disilicate trial illustrates [18]. This review does not permit comparative conclusions about clinical superiority across material systems.

### 4.2. Retention as a System-Level Outcome

Retention is a property of the complete system. Polished zirconia may show lower direct frictional interaction than some metallic pairs under particular conditions [8,42,56], but the included reports do not establish a universal zirconia-versus-metal hierarchy. Primary and secondary materials, taper, fit, surface condition, manufacturing method, lubrication, cyclic aging, and auxiliary mechanisms interact [6,7,8,24,28,29,33,34,35,36,37,38,39,40,41,42]. Seating force and the measurement protocol also affect the observed removal force. The results from one configuration cannot be transferred to another on the basis of the primary-material label alone.

Majcher et al. [57] described a dedicated cyclic testing method and illustrated it with a three-element telescopic crown comprising a CAD/CAM zirconia primary element, a galvanically produced gold intermediate component, and a cast CoCr external component. The report illustrates how test method and measurement conditions influence the interpretation of retention behavior. Numerical force endpoints presented graphically were not estimated for the present evidence map.

This distinction is clinically relevant because apparently similar zirconia primary crowns may behave differently when paired with electroformed gold, CoCr, zirconia, PEEK, PEKK, or FRC secondary components. A material label should therefore not substitute for the specification of the complete design and manufacturing protocol.

### 4.3. Interpretation of Auxiliary Retention-Control Mechanisms

Auxiliary elements serve distinct mechanical purposes. Friction pins and intermediate elements may generate or stabilize retention in selected designs, whereas occlusal stops primarily limit excessive loosening or removal forces [11,12,13,47]. Arnold’s comparison changed secondary material and fit concept as well as the presence of pins, so it did not isolate the pin effect [11]. Kochanek-Leśniewska et al. did not establish a clear matched retention advantage of gold over PMMA intermediate elements [12]. These findings should be described as design-specific engineering observations.

Laboratory evidence documents how these elements can alter force control within selected systems. No included clinical study evaluated their effects in isolation. This review therefore cannot infer that they improve prosthesis survival, reduce complications, or lower maintenance requirements. Future clinical evaluations should separate force regulation from patient handling and longer-term biological and technical outcomes.

### 4.4. Clinical Interpretation and Research Priorities

Available clinical evidence is concentrated on zirconia primary crowns combined with electroformed or electroplated gold secondary copings. Polymeric secondary structures appear in two additional one-year clinical trials, but polymeric primary crowns, other ceramic combinations, and auxiliary retention-control mechanisms remain predominantly laboratory topics. Clinical interpretation should retain the complete system specification and the distinction between short-term force or radiographic outcomes and longer-term survival, complications, and maintenance.

The small number of eligible clinical cohorts should not be interpreted as evidence that natural tooth double-crown rehabilitation is uncommon in practice. This review cannot determine whether the limited cohort-level evidence reflects limited prospective investigation, the publication of experience primarily as individual case reports, the predefined language and temporal restrictions, or broader reporting patterns. Because case reports were outside the scope, isolated demonstrations of feasibility are not represented. Future clinical studies should specify both crown materials, nominal and measured taper, fit, surface treatment, fabrication, initial and aged retention, prosthesis and abutment survival, complications, retention adjustments, maintenance, and patient-reported outcomes. Studies of auxiliary elements need comparisons that isolate the element itself. Tooth-only, combined tooth–implant, and implant-only outcomes should be separated, and linked reports should identify their shared cohort.

Beyond the mechanical outcomes mapped in this review, prosthodontic material and support selection also have broader biological and biomechanical dimensions. Preliminary in vitro work has reported composition-dependent cytotoxic and genotoxic responses to Ni-Cr dental alloys, with greater effects for a beryllium-containing formulation [58]. Finite element modeling has also been used to examine masticatory load-induced stress distributions around dental implants [59]. The earlier dental biomaterial literature has considered mercury exposure from dental amalgam in relation to biocompatibility and environmental impact [60]. In implant-supported rehabilitation, a pilot clinical study reported associations between peri-implant marginal bone loss and selected host-response markers, including IL-8 and total antioxidant status [61]. These observations concern broader dental biomaterials and implant biology and fall outside the present eligibility framework; they should not be interpreted as comparative evidence for the telescopic systems mapped here.

### 4.5. Limitations

The evidence base remains predominantly laboratory-based. Protocols differed in material pairing, taper definition, fit, surface treatment, seating load, withdrawal speed, lubrication, and aging. Some numerical results were available only graphically, and several reports contained internal inconsistencies. No values were estimated from graphs, and no quantitative pooling was performed. No formal critical appraisal was undertaken; consequently, the map does not establish the certainty of effects. Laboratory findings for auxiliary retention-control elements cannot be translated directly into clinical survival, complication rates, or maintenance burden. Pooled mixed-support outcomes also cannot be treated as tooth-only estimates. The restriction to English-language reports may have excluded relevant evidence. This limitation may be particularly relevant to telescopic and double-crown prosthodontics because a substantial part of the literature originates from non-English-speaking countries. The language restriction was predefined. Separately, relevant pre-2015 evidence lay outside the prespecified CAD/CAM-era temporal boundary. Two potentially relevant reports from the structured database search could not be retrieved despite additional retrieval attempts and therefore could not undergo full-text eligibility assessment. The backward reference-list check was limited to backward citation searching and did not include forward citation tracking or a new systematic database search. Database terminology was not fully harmonized, and semantic ranking in Elicit did not provide an independent human screening process.

## 5. Conclusions

Zirconia is the non-metal primary material represented the most often in the available clinical literature. Clinical reports are concentrated on zirconia with electroformed or electroplated gold secondaries, with two additional short-term cohorts examining polymeric secondaries. Clinical evidence remains very limited. Polymeric primary crowns and auxiliary retention-control elements were represented by laboratory evidence, and no clinical report isolated the auxiliary mechanisms. Retention depends on the complete material and design system, not the primary material alone. Mixed-support outcomes should not be generalized to tooth-only rehabilitation without subgroup data. This descriptive evidence map does not establish material or treatment superiority.

## Figures and Tables

**Table 1:** The distribution of the 48 included reports by evidence category. The seven clinical reports represent six identifiable cohorts; two Schwindling reports arise from the same randomized cohort.

Evidence Category	Reports	Main Role in Synthesis
Clinical	7 reports; 6 identifiable cohorts	Clinical retention, survival, complications, maintenance, radiographic changes, and patient-reported outcomes
In vitro	40	Retention/removal force, material pairing, taper, fit, wear, fracture, strain, and aging
In silico	1	Stress distribution in a tooth-supported telescopic crown model
Total	48	Descriptive evidence map; no formal critical appraisal or pooling

**Table 2:** Clinical evidence only: seven reports from six identifiable cohorts. Primary and secondary materials are listed separately. Pooled mixed-support outcomes are indirect for natural tooth rehabilitation when subgroup data are unavailable.

Cohort/Report	Design and Support	Sample/Prostheses	Primary/Secondary System	Follow-Up	Main Clinical Outcomes	Applicability/Limitations
Schwindling et al. (2017), two reports [16,17]	Randomized trial; tooth-supported	56 participants; 60 prostheses, 30 per group	Zirconia or CoCr primary; electroplated gold secondary; 1°	12 months (OHIP); 36 months (clinical)	Notably, 96.4% prosthesis survival in each group at 3 years; substantial aftercare. OHIP-49 improved, without clear material-group difference at 12 months.	One shared cohort, not two independent trials. Total of 54/60 prostheses re-examined at 36 months; survival denominator 28 per group.
Zahn et al. (2016) [18]	Prospective randomized trial; mixed tooth/implant support	29 patients; 37 prostheses; 77 teeth and 88 implants	Lithium-disilicate primary (IPS Empress 2); electroplated gold (27 prostheses) or Vectris FRC (10)	Mean 91 ± 57 months; maximum 173 months	Repairs in 88.9% of gold and 90% of Vectris prostheses; primary-crown stability concerns; unfavorable long-term Vectris outcomes.	Small, unequal groups. Most prosthesis outcomes pooled across support types; cannot supply tooth-only estimates.
Brandt et al. (2018) [19]	Retrospective patient survey; mixed support	74 patients; 87 prostheses: 22 tooth-only, 17 combined, 48 implant-only	Ceramic primary; electroplated gold secondary	Up to 16 years	High reported satisfaction; overall VAS approximately 88–90%.	Outcomes separated by jaw, not support type. Indirect for natural tooth rehabilitation; overlap with the 2019 dataset unresolved.
Brandt et al. (2019) [9]	Retrospective cohort; mixed support	111 patients; 126 prostheses: 19 tooth-only, 30 combined, 77 implant-only	Zirconia primary; electroformed gold secondary; 2°	Mean 45.25 months; up to 11.5 years	Pooled 5-year survival 96.9%; 5/6 failures in tooth-only subgroup (5/19, 26.3%). Combined subgroup: 100% survival, mean 44.48 months.	Small tooth-only subgroup. Pooled outcomes include implant-only prostheses; subgroup and pooled endpoints must remain distinct.
Refai et al. (2021) [20]	Randomized trial; tooth-supported maxillary Kennedy I	18 participants; 9 per group; no reported dropout	Zirconia primary; milled PEKK or PEEK secondary/framework	1 year	Dislodging force (mean ± SD, N): PEKK 19.52 ± 0.88 to 25.02 ± 2.76; PEEK 18.67 ± 0.53 to 20.10 ± 2.45.	Short-term whole-prosthesis force, not long-term survival. Published change row is inconsistent with endpoints; no change estimate used.
Elsarrif et al. (2021) [21]	Randomized trial; tooth-supported mandibular Kennedy I	14 participants; 7 per group	Zirconia primary (0° mesiodistal, 3° buccolingual); milled or pressed Bio-HPP secondary/framework	12 months	Reported 12-month abutment bone-height loss: 0.87 ± 0.13 vs. 0.45 ± 0.07 mm; residual ridge: 0.56 ± 0.08 vs. 0.75 ± 0.11 mm (milled vs. pressed).	Small short-term radiographic study. No quantitative retention or comparative long-term survival outcome.

## Data Availability

The original contributions presented in this study are included in the article and Supplementary Material. Further inquiries can be directed to the corresponding authors. A written protocol existed before screening but was not prospectively registered.

## Supplementary Materials

The following supporting information can be downloaded at https://www.mdpi.com/article/10.3390/dj14100687/s1. Table S1: Detailed report-level evidence map; Table S2: Funding and material-support statements; Table S3: Quantitative retention outcomes and principal testing conditions of laboratory studies reporting retention; File S1: Completed PRISMA-ScR Checklist. All references cited in Table S1–S3 are included in the main manuscript reference list [7,8,9,10,11,12,13,16,17,18,19,20,21,22,23,24,25,26,27,28,29,30,31,32,33,34,35,36,37,38,39,40,41,42,43,44,45,46,47,48,49,50,51,52,53,54,55,57]. Complete database search strategies are presented in Appendix A (Table A1).

## Abbreviations

The following abbreviations are used in this manuscript:

Abbreviation	Definition
CAD/CAM	Computer-aided design and computer-aided manufacturing
Ce-TZP/Al_2_O_3_	Ceria-stabilized tetragonal zirconia polycrystal/alumina nanocomposite
CoCr	Cobalt-chromium
EBSCO	Elton B. Stephens Company
FEA	Finite element analysis
FRC	Fiber-reinforced composite
N	Newton
OHIP-49	Oral Health Impact Profile-49
PEEK	Polyetheretherketone
PEKK	Polyetherketoneketone
PRISMA	Preferred Reporting Items for Systematic Reviews and Meta-Analyses
PRISMA-ScR	Preferred Reporting Items for Systematic Reviews and Meta-Analyses extension for Scoping Reviews
RCT	Randomized clinical trial
SLM	Selective laser melting
SLS	Selective laser sintering
UAKP	Unfilled aryl ketone polymer
Y-TZP	Yttria-stabilized tetragonal zirconia polycrystal
ZrO2	Zirconium dioxide

## Appendix A. Complete Search Strategies

All four structured database searches were run on 20 June 2026 with English-language and publication-year limits from 2015 onward. The original strings below are reproduced as actually run. Database syntax and material terminology differed and were not retrospectively harmonized. Elicit was used on 3 May 2026 for supplementary citation discovery and relevance triage of the first 1000 ranked records; it added no unique included report beyond the structured searches. The targeted backward reference-list check was a separate citation-searching route and did not constitute a rerun of these searches.

**Table A1:** Complete structured database search strategies and supplementary semantic-search query.

Source	Search Type	Search String or Query	Records
PubMed/MEDLINE	Structured database search	((telescop*[tiab] OR “telescopic crown”[tiab] OR “telescopic crowns”[tiab] OR “telescopic denture”[tiab] OR “telescopic dentures”[tiab] OR “telescopic overdenture”[tiab] OR “telescopic overdentures”[tiab] OR “double crown”[tiab] OR “double crowns”[tiab] OR “double-crown”[tiab] OR “double-crowns”[tiab] OR “cone crown”[tiab] OR “cone crowns”[tiab] OR “conical crown”[tiab] OR “conical crowns”[tiab] OR “Konus crown”[tiab] OR “Konus crowns”[tiab] OR “taper crown”[tiab] OR “taper crowns”[tiab] OR “tapered crown”[tiab] OR “tapered crowns”[tiab]) AND (zirconia[tiab] OR zirconium[tiab] OR ceramic*[tiab] OR alumina[tiab] OR “lithium disilicate”[tiab] OR PEEK[tiab] OR polyetheretherketone[tiab] OR PEKK[tiab] OR polymer*[tiab] OR resin*[tiab] OR composite*[tiab] OR “metal-free”[tiab] OR “non-metal”[tiab] OR “non-metallic”[tiab] OR nonmetal*[tiab]) AND (denture*[tiab] OR overdenture*[tiab] OR prosthes*[tiab] OR prosthodontic*[tiab] OR dental[tiab]))	127
Scopus	Structured database search	TITLE-ABS-KEY ((telescop* W/3 (crown* OR coping* OR denture* OR overdenture* OR prosthes* OR attachment* OR retainer*) OR “telescopic crown*” OR “telescopic denture*” OR “telescopic overdenture*” OR “double crown*” OR “double-crown*” OR “cone crown*” OR “conical crown*” OR “Konus crown*” OR “taper crown*” OR “tapered crown*”) AND (zirconia OR zirconium OR ceramic* OR alumina OR “lithium disilicate” OR PEEK OR polyetheretherketone OR PEKK OR polymer* OR resin* OR composite* OR “metal-free” OR “non-metal*” OR nonmetal*) AND (denture* OR overdenture* OR prosthes* OR prosthodontic* OR dental))	147
Web of Science	Structured database search	TS=((telescop* NEAR/3 (crown* OR coping* OR denture* OR overdenture* OR prosthes* OR attachment* OR retainer*) OR “telescopic crown*” OR “telescopic denture*” OR “telescopic overdenture*” OR “double crown*” OR “double-crown*” OR “cone crown*” OR “conical crown*” OR “Konus crown*” OR “taper crown*” OR “tapered crown*”) AND (zirconia OR zirconium OR ceramic* OR alumina OR “lithium disilicate” OR PEEK OR polyetheretherketone OR PEKK OR polymer* OR resin* OR composite* OR “metal-free” OR “non-metal*” OR nonmetal*) AND (denture* OR overdenture* OR prosthes* OR prosthodontic* OR dental))	149
Dentistry & Oral Sciences Source (EBSCO)	Structured database search	(TI (telescop* OR “telescopic crown*” OR “telescopic denture*” OR “telescopic overdenture*” OR “double crown*” OR “double-crown*” OR “cone crown*” OR “conical crown*” OR “Konus crown*” OR “taper crown*” OR “tapered crown*”) OR AB (telescop* OR “telescopic crown*” OR “telescopic denture*” OR “telescopic overdenture*” OR “double crown*” OR “double-crown*” OR “cone crown*” OR “conical crown*” OR “Konus crown*” OR “taper crown*” OR “tapered crown*”)) AND (TI (zirconia OR zirconium OR ceramic* OR alumina OR “lithium disilicate” OR PEEK OR polyetheretherketone OR PEKK OR polyetherketoneketone OR polymer* OR resin* OR composite* OR “metal-free” OR “non-metal*” OR “nonmetal*”) OR AB (zirconia OR zirconium OR ceramic* OR alumina OR “lithium disilicate” OR PEEK OR polyetheretherketone OR PEKK OR polyetherketoneketone OR polymer* OR resin* OR composite* OR “metal-free” OR “non-metal*” OR “nonmetal*”)) AND (TI (denture* OR overdenture* OR prosthes* OR prosthodontic* OR dental OR “removable partial denture*” OR “removable dental prosthes*”) OR AB (denture* OR overdenture* OR prosthes* OR prosthodontic* OR dental OR “removable partial denture*” OR “removable dental prosthes*”))	121
Elicit (Semantic Scholar and OpenAlex)	Supplementary semantic search	In telescopic or double-crown removable dental prostheses, how do non-metal primary copings, with or without auxiliary retention attachments, perform in terms of retention, survival, wear, complications, and maintenance?	1000 ranked records; no unique included reports
