# Supplementary material for: Non-Metal Primary Crowns in Telescopic and Double-Crown Systems Involving Natural Teeth: A Scoping Review
Source: Dent J (Basel). 2026 Oct 21;14(10):687. doi: 10.3390/dj14100687 (PMC13703499; doi:10.3390/dj14100687)
Supplement: Supplementary file 1 [file dentistry-14-00687-s001.zip › Tables_r1_clean.pdf]

Supplementary Material

Non-Metal Primary Crowns in Telescopic and Double-Crown Systems Involving Natural Teeth: A Scoping Review

Supplementary Table S1. Detailed Report-Level Evidence Map

The map contains 48 reports: 40 in vitro, one in silico, and seven clinical reports representing six identifiable cohorts. S1 IDs 7 and 8 arise from the same Schwindling randomized cohort. Other possible dataset overlaps are identified where unresolved. Report IDs are stable identifiers for this supplement; bracketed references correspond to the manuscript. Indirect crown-pair engineering evidence concerns the crown interface on a standardized substrate and does not establish clinical transferability.

ID	Report	Design and support	Materials, geometry and sample	Retention-control element	Aging or follow-up	Principal findings and qualifications
1	Arnold et al. (2018) [11]	In vitro; Germany; direct tooth-supported laboratory model	Primary: zirconia in groups C and E. Secondary: electroplated gold in group C; CoCr with gold-alloy friction pins in group E. Geometry: 2° (n = 5 systems/group).	Present only in group E: clearance fit with gold-alloy friction pins.	15,000 axial insertion/removal cycles; 50,000 non-axial cycles in total (20,000 before and 30,000 after axial cycling)	Group E changed from 12.2 to 12.4 N; group C lost 39% (7.4 to 4.5 N). The comparison is confounded by different secondary materials and fit concepts, so it does not isolate a pin effect.
2	Kochanek-Leśniewska et al. (2017) [12]	In vitro; Poland; indirect crown-pair engineering evidence	Primary: zirconia in five test groups; gold-alloy control. Intermediate: electroformed gold or milled PMMA. External: zirconia, CoCr, or acrylic. Geometry: 1°; 18 systems (n = 3/group).	Present: intermediate retention element/electroformed or CAD/CAM	Up to 30,000 simulated load cycles	Groups A and B were most stable; A vs B and C vs D did not differ significantly. One control lost retention at 16,800 cycles. The E1 run ended at 10,000 cycles because the lower holder post fractured, not because a crown fractured. Table 1 labels the external alloy CrCo but names WIRON 88; the source alloy description is inconsistent.
3	Schwindling et al. (2017) [47]	In vitro; Germany; indirect crown-pair engineering evidence	Primary: Zirconia. Secondary: Zirconia. Taper/geometry: 3° and 4° conical crowns	Present: zirconia or composite occlusal stops	Retention-adjustment testing; cycling not central/not reported	Zirconia and composite stops capped mean maximum loosening forces at 12.1 and 12.8 N, respectively; the stops limited excessive retention rather than increasing it.
4	Tasaka et al. (2022) [13]	In vitro; Germany/Japan; indirect crown-pair engineering evidence	Primary: zirconia. Secondary: FRC. Geometry: 3°; 32 secondary crowns (n = 16/polishing group).	Present: composite resin occlusal stop	10,000 cycles	Composite stops limited excessive removal forces. FRC mean ± SD increased from 16.12 ± 5.92 to 21.61 ± 8.82 N. The separately reported mean change is inconsistent with these endpoints; see S3.
5	Brandt et al. (2018) [19]	Retrospective survey; Germany; mixed support; 74 patients/87 prostheses	Ceramic primary crowns and electroplated secondary copings; 22 tooth-supported, 17 combined tooth-implant-supported, and 48 implant-supported prostheses.	Absent	Up to 16 years	High pooled patient satisfaction (overall VAS approximately 88%-90%). Outcomes were reported by jaw location, not support type; applicability to natural-tooth systems is therefore indirect.

ID	Report	Design and support	Materials, geometry and sample	Retention-control element	Aging or follow-up	Principal findings and qualifications
6	Brandt et al. (2019) [9]	Retrospective cohort; Germany; mixed support; 111 patients/126 prostheses	Zirconia primary crowns/electroformed gold; 2°. Support: 19 tooth-only, 30 combined tooth-implant, 77 implant-only prostheses.	Absent	Mean 45.25 months; up to 11.5 years	Overall 5-year survival was 96.9%. Five of six failures occurred in the tooth-only subgroup (26.3% subgroup failure); combined-support survival was 100% over mean 44.48 months.
7	Schwindling et al. (2017) [17]	Randomized clinical trial report; Germany; tooth-supported; 56 participants/60 prostheses; same randomized cohort (S1 IDs 7 and 8)	Primary: Zirconia vs CoCr. Secondary: Electroplated gold. Taper/geometry: 1°	Absent	36 months	Prosthesis survival was 96.4% at three years in both groups; technical aftercare was common. This is the clinical outcome report from the same cohort as ID 8.
8	Schwindling et al. (2017) [16]	Randomized clinical trial report; Germany; tooth-supported; 56 participants/60 prostheses; same randomized cohort as S1 ID 7	Primary: Zirconia vs CoCr. Secondary: Electroplated gold. Taper/geometry: 1°	Absent	12 months	OHIP-49 improved after treatment, without a clear material-group difference at 12 months. This is the patient-reported outcome report from the same cohort as ID 7.
9	Zahn et al. (2016) [18]	Prospective randomized trial; Germany; mixed support; 29 patients/37 prostheses	IPS Empress 2 lithium-disilicate primary crowns; Vectris FRC or electroplated-gold secondaries. Support: 77 natural teeth and 88 implants among 165 abutments; support-specific prosthesis outcomes unavailable.	Absent	Mean 91 ± 57 months; maximum 173 months	Repairs occurred in 88.9% of electroplated-gold and 90% of Vectris prostheses. The report described primary-crown stability concerns and unfavorable long-term Vectris outcomes. Most prosthesis outcomes were pooled across support types.
10	Schwindling et al. (2016) [25]	In vitro; Germany; indirect crown-pair engineering evidence	Primary: Zirconia. Secondary: Zirconia. Taper/geometry: 1° and 2°	Absent	50,000 cycles	Retention after 50,000 cycles was described as stable within the tested all-zirconia configurations; exact group endpoints were not extractable from the graphs. See S3.
11	Merk et al. (2016) [24]	In vitro; Germany; indirect crown-pair engineering evidence	Primary: Y-TZP and CoCr. Secondary: Y-TZP; electroformed gold comparator noted. Taper/geometry: 0°, 1°, 2°	Absent	20 pull-off cycles	Retention depended on both taper and primary/secondary material combination. Taper did not produce a uniform effect across all groups; see S3.
12	Merk et al. (2016) [7]	In vitro; Germany; indirect crown-pair engineering evidence	Primary: Zirconia. Secondary: PEEK. Taper/geometry: 0°, 1°, 2°	Absent	20 pull-off cycles	Fabrication method and taper influenced retention of PEEK secondary crowns on zirconia primaries.
13	Luft et al. (2021) [23]	In vitro; Germany; indirect crown-pair engineering evidence	Primary: Zirconia, gold, CoCr. Secondary: Galvanoformed gold, CoCr, PEEK. Taper/geometry: 0° (parallel primary crowns)	Absent	10,000 cycles	Retention-force behavior differed by material pairing; zirconia/PEEK and zirconia/galvanoformed combinations showed different retention-loss patterns.

ID	Report	Design and support	Materials, geometry and sample	Retention-control element	Aging or follow-up	Principal findings and qualifications
14	Igarashi et al. (2023) [27]	In vitro; Japan/Switzerland; indirect crown-pair engineering evidence	Primary: Zirconia, titanium, CoCr, PEKK, gold. Secondary: PEKK; galvano-gold control. Taper/geometry: 2°	Absent	20,000 cycles	PEKK/PEKK and Au/PEKK retention increased, while zirconia/PEKK decreased after cycling.
15	Priester et al. (2021) [33]	In vitro; Germany; indirect crown-pair engineering evidence	Primary: PEEK, zirconia, CoCr. Secondary: PEEK. Taper/geometry: 0°	Absent	10,000 cycles	PEEK/PEEK was described as comparatively constant over cycling within this experiment. Force levels also depended on direction of withdrawal; long-term endpoints were graphical.
16	Stock et al. (2016) [28]	In vitro; Germany/Switzerland; indirect crown-pair engineering evidence	Primary: PEEK. Secondary: CoCr, zirconia, or electroformed gold. Geometry: 0, 1, or 2°; 90 systems.	Absent	20 pull-off cycles	Retention varied by secondary material and taper; electroformed-gold groups were lower at 1 and 2°.
17	Stock et al. (2016) [29]	In vitro; Germany/Switzerland; indirect crown-pair engineering evidence	Primary and secondary: PEEK. Secondary crowns were milled or pressed from pellets or granules. Geometry: 0, 1, or 2°; 90 systems.	Absent	20 pull-off cycles	Fabrication method and taper interacted; no uniform taper effect was observed across fabrication routes.
18	Nakamura et al. (2021) [31]	In vitro; Japan; indirect crown-pair engineering evidence	Primary/secondary: PEEK/PEEK or type-4 gold/gold. Geometry: 6°; seven systems/group.	Absent	10,000 cycles	The report gives approximate initial forces of 12 N and approximately 6.5 N for PEEK after 10,000 cycles. The reported approximate values are retained as approximate; graph values were not estimated.
19	Schimmel et al. (2021) [34]	In vitro; Switzerland; indirect crown-pair engineering evidence	Primary: zirconia, titanium, PEEK, non-precious alloy, precious alloy. Secondary: PEEK, non-precious alloy, precious alloy. Taper/geometry: 1°	Absent	10,000 cycles	Retention force changes depended on CAD/CAM material combinations.
20	Micovic Soldatovic et al. (2023) [43]	In vitro; Germany; indirect crown-pair engineering evidence	Primary: CoCr or zirconia. Secondary: PEEK, PEKK, CoCr, or zirconia; four polymer groups used 0.5-mm secondaries bonded to CoCr tertiary structures. Geometry: 0°; 12 groups, n = 15/group.	Tertiary CoCr crown in some groups; not a classic auxiliary insert	Up to 10,000 cycles	Polymer secondaries on CoCr/zirconia primaries tended to increase retention after cycling, while some rigid pairings decreased.
21	Awad et al. (2025) [22]	In vitro; Egypt; combined natural-tooth/implant laboratory model	Matched primary/secondary pairs of zirconia, PEKK, or CoCr in models with two canines and two implants. Eighteen models, six per group.	Absent	1,440 cycles	Retention decreased in all groups after 1,440 cycles; PEKK remained higher than zirconia.
22	Mostafa et al. (2021) [30]	In vitro; Egypt; direct tooth-supported laboratory model	Primary: PEKK or zirconia (24 each). Secondary/framework: PEKK; taper not reported. Forty-eight primary crowns (24 per material) were assembled in tooth-supported prosthesis models; repeated pull-offs are not independent specimens.	Absent	6 pull-offs/specimen	PEKK/PEKK showed higher initial friction fit than zirconia/PEKK in the extracted data.

ID	Report	Design and support	Materials, geometry and sample	Retention-control element	Aging or follow-up	Principal findings and qualifications
23	Mohamed et al. (2022) [54]	In vitro; Egypt; direct tooth-supported laboratory model	Primary: PEKK or zirconia (four/cast). Secondary/framework: PEKK; 2° taper. Two casts and eight primary crowns, four per cast.	Absent	Not specified	Zirconia primary crowns produced higher measured strain than PEKK under bilateral and unilateral loading.
24	Mendes Tribst et al. (2022) [53]	In silico; tooth-supported finite element model	Primary: Zirconia, CoCr, titanium, PEKK. Secondary: PEKK. Taper/geometry: 6°	Absent	Not applicable	Assigned primary-material elastic modulus affected secondary-crown and cement-layer stress within the modeled conditions. These computational results do not establish clinical failure risk.
25	Pott et al. (2025) [48]	In vitro; Germany; indirect crown-pair engineering evidence	Primary/secondary: CoCr/CoCr, CoCr/PEEK, PEEK/PEEK, zirconia/PEEK, or zirconia/zirconia; n = 40/group.	Absent	10,000 cycles	Wear behavior differed by material pairing; zirconia/PEEK was reported as a low-wear pairing.
26	Muwafi et al. (2022) [50]	In vitro; Egypt; direct tooth-supported laboratory model	Primary crowns and telescopic frameworks: CoCr or Bio-HPP; digitally designed. Twelve prostheses, six per material.	Absent	50,000 chewing cycles	Bio-HPP showed higher wear than CoCr in the extracted data.
27	Donia et al. (2022) [26]	In vitro; Egypt; indirect crown-pair engineering evidence	Primary/secondary: PEEK/PEEK; 2- or 6° taper; 1- or 2-mm secondary thickness.	Absent	Up to 1,440 cycles / simulated months	The Methods and Table 2 describe PEEK/PEEK in all four taper/thickness groups, with retention loss over cycling. The abstract contains inconsistent material and retention descriptions; the tabulated data were used.
28	Yoshikawa et al. (2019) [37]	In vitro; Japan; indirect crown-pair engineering evidence	Primary: Ce-TZP/Al2O3 zirconia/alumina. Secondary: Ce-TZP/Al2O3 zirconia/alumina. Taper/geometry: 2° and 4°	Absent	10,000 insertion/removal cycles	2° crowns lost less retention than 4° crowns over cycles; taper strongly affected retention.
29	Fischer et al. (2020) [8]	In vitro; Romania; indirect crown-pair engineering evidence	Primary: Zirconia, CoCr. Secondary: CoCr. Taper/geometry: 0° and 2°. Fifteen systems per material/taper group; three were assigned to each measurement interval.	Absent	Up to 360 cycles	Zirconia-based telescopic systems showed higher retention than CoCr-based systems at 0° and 2°.
30	Kawilarang et al. (2020) [36]	In vitro; Indonesia; indirect crown-pair engineering evidence	Matched primary/secondary CoCr or zirconia pairs; geometry: 0, 1, or 2°. Twenty-four systems, four per group.	Absent	Not reported	0° taper showed highest retention; zirconia at 0° reported higher retention than CoCr at 2°.
31	Çelik Güven et al. (2017) [44]	In vitro; Turkey; indirect crown-pair engineering evidence	Primary: Zirconia, gold, CoCr, laser-sintered alloy, titanium. Secondary: Electroformed and matched alloys. Taper/geometry: 4° and 6°	Absent	5,000 cycles	Retention and wear behavior varied by fabrication method, material, and taper.

ID	Report	Design and support	Materials, geometry and sample	Retention-control element	Aging or follow-up	Principal findings and qualifications
32	Goguță et al. (2021) [45]	In vitro; Romania; indirect crown-pair engineering evidence	Primary: CAD/CAM zirconia or SLS/SLM metal; metal secondary crowns; 6° taper. Table 2 summarizes 84 observations, seven per tooth/material/wet-dry condition. Methods also describes 14 crowns per tooth/material set; these are not 84 independently aged assemblies.	Absent	Not reported	Retention differed with tooth form, fabrication, and lubrication. Table 1 and narrative descriptions of the metal alloy are inconsistent; the source labels are preserved and flagged.
33	Abdallah et al. (2023) [46]	In vitro; Egypt; direct tooth-supported laboratory model	Primary: zirconia or CoCr; secondary/framework: CoCr. Fourteen sets of primary crowns and corresponding removable partial dentures, seven sets per material group; not 14 individual crowns. Marburg design, 1.5–2 mm parallel cervical band and 2°–4° occlusal taper.	Absent	50,000 chewing cycles + 270 insertion/removal cycles	Zirconia primary crowns initially and finally retained higher than CoCr, although both decreased after aging.
34	Milić-Lemić et al. (2020) [49]	In vitro; Serbia; indirect crown-pair engineering evidence	Primary: zirconia; secondary: milled PEEK, or electroplated gold coping with a zirconia tertiary structure. Ten specimens per group; no PEEK tertiary component in the gold group.	Absent	800-900 cycles	Early retention settling was described in both systems. Reported means reached 3.0 N for zirconia/PEEK and 4.1 N for zirconia/gold with zirconia tertiary after 900 cycles. The source conclusion about an optimal 5–9 N range does not match these endpoint means.
35	Tasaka et al. (2022) [10]	In vitro; Germany/Japan; indirect crown-pair engineering evidence	Primary: Zirconia. Secondary: Fiber-reinforced composite. Taper/geometry: 0°	Absent	10,000 cycles	Zirconia/FRC crowns maintained retention in selected milling/polishing conditions.
36	Kotthaus et al. (2019) [32]	In vitro; Germany; indirect crown-pair engineering evidence	Primary: gold alloy, non-precious alloy, zirconia, or PEKK. Secondary: PEKK. Geometry: 0°.	Absent. Grub screws were used only to mount secondary crowns in the wear simulator.	10,000 cycles	Retention increased during the first 2,000 cycles and then stabilized; zirconia showed the least primary-crown wear. The abstract states 5,000 cycles, while Methods and Results describe 10,000; the latter were used.
37	Melitt et al. (2025) [51]	In vitro; India; direct tooth-supported laboratory model	Matched primary/secondary PEEK or zirconia systems; 6° convergence. Twenty tooth-supported epoxy models, ten per group.	Absent	Thermocycling; fracture-load testing	Zirconia showed higher fracture load; PEEK showed more repairable failure patterns.
38	Lümkemann et al. (2020) [52]	In vitro; Germany; indirect crown-pair engineering evidence	Primary: Zirconia. Secondary: UAKP/high-performance aryl ketone polymer. Taper/geometry: 0°	Absent	1.2 million chewing cycles + thermocycling	Veneered high-performance aryl-ketone polymer secondary crowns on zirconia primaries underwent fracture testing after aging. Fracture loads describe the tested assemblies and are not clinical survival estimates.

ID	Report	Design and support	Materials, geometry and sample	Retention-control element	Aging or follow-up	Principal findings and qualifications
39	Enaba (2022) [38]	In vitro; Egypt; indirect crown-pair engineering evidence	Primary and secondary: PEEK or graphene-reinforced PMMA; geometry: 0, 1, or 2°.	Absent	20 cycles	Retention decreased with increasing taper; PEEK at 0° showed higher values than at 2°.
40	Linek et al. (2016) [35]	In vitro; Germany; indirect crown-pair engineering evidence	Lithium-disilicate primary (IPS Empress 2)/electroformed-gold secondary; five metal-primary comparator groups; 0°; ten pairs per group.	Absent	10,000 insertion/removal cycles; distilled water, 36°C	Eligible ceramic group: mean ± SD 3.4 ± 1.1 to 2.9 ± 0.8 N; median 3.1 to 2.8 N. Table 4 reports −10% median change; its prose statement of 75% remaining retention is inconsistent.
41	Nakagawa et al. (2017), Dental Materials Journal [39]	In vitro; Japan; indirect crown-pair engineering evidence	Ce-TZP/Al2O3 primary and secondary crowns; 2°, 4°, or 6° taper; 0 or 10 µm space. One primary per taper and five secondaries per taper/space combination (30 secondaries).	Absent	Repeated pull-offs under 50 or 100 N seating; no aging protocol	Taper and seating force affected retention; 6° groups were described as having no retention. Selected taper-level means were given, but exact gap-specific means were graphical. See S3.
42	Nakagawa et al. (2017), Journal of Osaka Dental University [40]	In vitro; Japan; indirect crown-pair engineering evidence	Ce-TZP/Al2O3 primary and secondary crowns; L18 Taguchi design, 18 condition combinations, eight factors including 2°, 4°, and 6° taper.	Absent	Repeated pull-offs under 50 or 100 N seating; no aging protocol	Reported factor-level retention estimates were 35.8, 15.9, and 1.4 N for 2°, 4°, and 6°, with a 99% confidence interval half-width of 6.6 N. These are factor summaries, not independent group means.
43	Nakajima et al. (2019) [41]	In vitro; Japan; indirect crown-pair engineering evidence	Ce-TZP/Al2O3 primary and secondary crowns; 2° or 4° half-angle; five pairs per taper, tested in dry and water conditions with 25, 50, or 100 N seating.	Absent	Repeated pull-offs; no cyclic aging; water 37°C or dry	Reported retention was greater in water than dry conditions for each matched taper/load comparison. Means are tabulated in S3; dispersion values were graphical.
44	Fujiki et al. (2020) [42]	In vitro; Japan; indirect crown-pair engineering evidence	Ce-TZP/Al2O3 telescopic pairs; 2° or 4° half-angle; five pairs per taper. Polished/non-polished, polished/blasted, or blasted/blasted surfaces; 25 or 50 N seating. Separate flat-coupon tests were also performed.	Absent	Repeated dry pull-offs; no cyclic aging	Crown-pair retention varied with taper, surface condition, and seating load. Flat-coupon friction outcomes are not conflated with telescopic removal force; S3 charts only the crown-pair outcomes.
45	Muwafi et al. (2022), strain report [55]	In vitro; Egypt; direct tooth-supported laboratory model	Maxillary Kennedy I model; 12 telescopic partial dentures, six each with matched CoCr or Bio-HPP primaries/secondaries. Marburg geometry: 1.5–2 mm parallel cervical band and 6° occlusal cone.	Absent	240,000 chewing cycles; static strain tests at 100 N	Strain distributions at abutment and ridge sites differed under bilateral and unilateral loads. No retention-force outcome was reported. These are model strain observations, not clinical bone or tooth outcomes.
46	Refai et al. (2021) [20]	Randomized clinical trial; Egypt; tooth-supported maxillary Kennedy I; 18 men, nine per group	Zirconia primary crowns; 1 mm parallel cervical band and 3° occlusal taper; milled PEKK or PEEK secondary/framework structures.	Absent	1 year; no reported dropout	Mean ± SD dislodging force: PEKK 19.52 ± 0.88 to 25.02 ± 2.76 N; PEEK 18.67 ± 0.53 to 20.10 ± 2.45 N. Between-group $p = 0.009$ at one year. The source change row is inconsistent with endpoints and is not used to estimate change.

ID	Report	Design and support	Materials, geometry and sample	Retention-control element	Aging or follow-up	Principal findings and qualifications
47	Elsarrif et al. (2021) [21]	Randomized clinical trial; Egypt; tooth-supported mandibular Kennedy I; 14 patients, seven per group	Zirconia primaries, 0.5 mm thickness; 0° mesiodistal and 3° buccolingual taper. Bio-HPP secondary/framework structures milled from blanks or pressed from granules; 35 µm space.	Absent	12 months	Mean ± SD bone-height loss for milled versus pressed frameworks: abutments 0.87 ± 0.13 versus 0.45 ± 0.07 mm; residual ridge 0.56 ± 0.08 versus 0.75 ± 0.11 mm. No numerical retention force was reported.
48	Majcher et al. (2017) [57]	In vitro; Poland; indirect crown-pair engineering evidence; methodological/device study	Illustrated three-element crown: CAD/CAM zirconia primary/inner element; galvanically produced gold intermediate component; cast Cr-Co external/female element. Other material combinations discussed included Au, ZrO2, Cr-Co, and PMMA. Sample size NR in the extractable text.	Present: three-element design with galvanic gold intermediate component	Cyclic connection/disconnection testing with a dedicated device; exact cycle count for the illustrated example NR in extractable text	The report described a dedicated method for measuring retention during cyclic wear simulation and illustrated stable and increasing retention-behavior patterns across crown designs. The eligible example contained a zirconia primary crown. Numerical force endpoints shown graphically were not estimated.

Abbreviations: CAD/CAM, computer-aided design/computer-aided manufacturing; Ce-TZP/Al2O3, ceria-stabilized zirconia/alumina nanocomposite; CoCr, cobalt-chromium alloy; FEA, finite element analysis; FRC, fiber-reinforced composite; NR, not reported or not extractable; OHIP, Oral Health Impact Profile; PEEK, polyetheretherketone; PEKK, polyetherketoneketone; PMMA, polymethyl methacrylate; RPD, removable partial denture; SD, standard deviation; SLM, selective laser melting; SLS, selective laser sintering; VAS, visual analogue scale; Y-TZP, yttria-stabilized tetragonal zirconia polycrystal. Bio-HPP denotes the source-reported PEEK-based material.

Supplementary Table S2. Funding and Material-Support Statements

Statements are charted descriptively and are not a risk-of-bias assessment. Not reported means that no relevant statement was located in the available report, not that support did not exist. Study funding, supplied materials, manufacturing or equipment assistance, and open-access publication funding are distinguished where stated.

S1 ID	Report	Funding and material or technical support
1	Arnold et al. (2018) [11]	Departmental support from the Department of Prosthodontics, Martin-Luther-University Halle-Wittenberg, Germany.
2	Kochanek-Leśniewska et al. (2017) [12]	No specific grant from public, commercial, or not-for-profit funding agencies.
3	Schwindling et al. (2017) [47]	Brasseler supplied custom 3° and 4° milling burs. One author received support from the University of Heidelberg Physician Scientist Programme. DeguDent zirconia products were used, but a donation of blanks was not stated.
4	Tasaka et al. (2022) [13]	No specific grant from public, commercial, or not-for-profit funding agencies. Shofu supplied FRC discs.
5	Brandt et al. (2018) [19]	Not reported.

S1 ID	Report	Funding and material or technical support
6	Brandt et al. (2019) [9]	Not reported.
7	Schwindling et al. (2017) [17]	Nobel Biocare Services AG grant 2005-362; one author received support from the University of Heidelberg Physician Scientist Programme.
8	Schwindling et al. (2017) [16]	Nobel Biocare Services AG grant 2005-362; Hans Plewe GmbH supported laboratory work. One author received support from the University of Heidelberg Physician Scientist Programme.
9	Zahn et al. (2016) [18]	Not reported.
10	Schwindling et al. (2016) [25]	DeguDent supplied semi-sintered zirconia blanks. One author received support from the University of Heidelberg Physician Scientist Programme.
11	Merk et al. (2016) [24]	Financial support from bredent; galvanic device supplied by C. Hafner; Ceramill materials supplied by Amann Girrbach.
12	Merk et al. (2016) [7]	Financial support from bredent; software support from Dental Softworks.
13	Luft et al. (2021) [23]	Materials supplied by C. Hafner and bredent; CAD/CAM manufacturing assistance from the Matthias Gürtler dental laboratory.
14	Igarashi et al. (2023) [27]	No study funding declared; open-access funding provided by the University of Bern. Daishin Trading and Wada Precision Dental Laboratories assisted with sample fabrication; Satista provided statistical assistance.
15	Priester et al. (2021) [33]	Not reported.
16	Stock et al. (2016) [28]	Financial support from bredent; galvanic device supplied by C. Hafner; Ceramill materials supplied by Amann Girrbach.
17	Stock et al. (2016) [29]	Materials and financial support from bredent; software-engineering support acknowledged.
18	Nakamura et al. (2021) [31]	Not reported.
19	Schimmel et al. (2021) [34]	Swiss Dental Association research funds supported the testing device. Bredent supplied the milled specimens; the University of Bern provided open-access funding.
20	Micovic Soldatovic et al. (2023) [43]	Bredent and Cendres+Métaux supplied materials. Open-access funding through Projekt DEAL; no separate study grant stated.
21	Awad et al. (2025) [22]	None.
22	Mostafa et al. (2021) [30]	Not reported.
23	Mohamed et al. (2022) [54]	Not reported.
24	Mendes Tribst et al. (2022) [53]	Princess Nourah bint Abdulrahman University Researchers Supporting Project PNURSP2022R6.
25	Pott et al. (2025) [48]	Xental Dentaltechnik assisted with design and manufacture; microscopy assistance from Hannover was acknowledged. Open-access funding through Projekt DEAL; no separate study grant stated.

S1 ID	Report	Funding and material or technical support
26	Muwafi et al. (2022) [50]	Not reported.
27	Donia et al. (2022) [26]	Not reported.
28	Yoshikawa et al. (2019) [37]	Not reported.
29	Fischer et al. (2020) [8]	No external funding. The Fischer Dental Laboratory assisted with specimen manufacture.
30	Kawilarang et al. (2020) [36]	Not reported.
31	Çelik Güven et al. (2017) [44]	DEN-TEK Dental and Dental Materials Inc. Co. assisted with specimen manufacture. No separate funding statement located.
32	Goguță et al. (2021) [45]	Laboratory assistance from Radent and Probis, Timișoara. No separate funding statement located.
33	Abdallah et al. (2023) [46]	No financial support.
34	Milić-Lemić et al. (2020) [49]	Dental Design Studio and Nemanja Grkinić assisted with specimen preparation; Gustav Bačlija (Gdent) provided the galvanic device. No separate funding statement located.
35	Tasaka et al. (2022) [10]	Supported by Shofu Inc., which supplied FRC discs.
36	Kotthaus et al. (2019) [32]	Research funding: none to report (explicit statement).
37	Melitt et al. (2025) [51]	Not reported.
38	Lümkemann et al. (2020) [52]	Materials and financial support from Solvay Dental 360.
39	Enaba (2022) [38]	Not reported.
40	Linek et al. (2016) [35]	DeguDent, Ivoclar Vivadent, and Wieland supplied materials without charge. Jenoptik/Hommelwerke assisted with surface roughness measurements; TU Dresden provided statistical assistance.
41	Nakagawa et al. (2017), Dental Materials Journal [39]	Osaka Dental University Research Funds, Encouraging Scientific Research grant 15-03 (FY2015).
42	Nakagawa et al. (2017), Journal of Osaka Dental University [40]	Osaka Dental University Encouraging Scientific Research grant 15-03 and Oral Implant Research grant 16-01.
43	Nakajima et al. (2019) [41]	Not reported. Departmental assistance was acknowledged, without a financial-support statement.
44	Fujiki et al. (2020) [42]	Not reported.
45	Muwafi et al. (2022), strain report [55]	The research did not receive financial support.
46	Refai et al. (2021) [20]	Source of support: Nil.

S1 ID	Report	Funding and material or technical support
47	Elsarrif et al. (2021) [21]	Not reported.
48	Majcher et al. (2017) [57]	Not reported in the accessible article text.

Supplementary Table S3. Quantitative Retention Outcomes and Principal Testing Conditions of Laboratory Studies Reporting Retention

Values are reproduced using the units and summary measures reported by the original studies. NR indicates that the numerical value or testing condition was not reported or was not extractable from the published report. Values were not estimated from graphical data.

This table contains 33 laboratory reports. Separate rows preserve experimental groups or, where explicitly labeled, factor-level summaries. Testing conditions apply to all rows within a report unless otherwise stated. Metallic comparator groups are retained within eligible reports. Repeated measurements are not interpreted as independent specimens. Numerical values were not estimated from graphs.

S1 ID 1: Arnold et al. (2018) [11]

Testing: insertion/removal cycles: 15,000 axial insertion/removal cycles at 10 mm/s; 20 N; chewing/mechanical loading: 50,000 non-axial cycles in total: 20,000 before and 30,000 after axial cycling; 50 N, 1 Hz; thermocycling: NR.

Conditions: Artificial saliva (Glandosane); 22 ± 2°C; withdrawal/crosshead speed: 50 mm/min; seating: 50 N. Summary measure: Mean ± SD.

Group / condition S1 ID 1	Primary material	Secondary material	Taper or geometry	Group n / unit	Initial or principal force	Final/post-aging force	Absolute or percentage change
A	High-noble alloy	High-noble alloy	0°	5 paired-crown systems	9.6 ± 4.4 N	2.9 ± 2.0 N	70% loss (reported)
B	CoCr	CoCr	0°	5 paired-crown systems	12.5 ± 5.1 N	4.6 ± 2.9 N	64% loss (reported)
C	Zirconia	Electroplated gold	2°	5 paired-crown systems	7.4 ± 5.1 N	4.5 ± 3.1 N	39% loss (reported)
D; friction pins	CoCr	CoCr; CoCr pins	2°; clearance fit	5 paired-crown systems	13.9 ± 5.7 N	10.0 ± 3.2 N	28% loss (reported)
E; friction pins	Zirconia	CoCr; gold-alloy pins	2°; clearance fit	5 paired-crown systems	12.2 ± 2.3 N	12.4 ± 3.5 N	+0.2 N; +1.6% (calculated from means)
F	High-noble alloy	High-noble alloy	6°	5 paired-crown systems	6.0 ± 2.4 N	3.2 ± 1.8 N	47% loss (reported)

Principal comparison: Kruskal–Wallis/Mann–Whitney U; initial-to-final change in group E was not significant.

Qualification: Forces concern paired crowns in a mandibular tooth-supported model. C versus E differs in secondary material and fit as well as pins; the pin effect is not isolated.

S1 ID 2: Kochanek-Leśniewska et al. (2017) [12]

Testing: insertion/removal cycles: Up to 30,000; five retention measurements after every 100 load cycles; chewing/mechanical loading: NR; thermocycling: NR.

Conditions: Dry; no lubricant used (Discussion, PDF p. 9); withdrawal/crosshead speed: NR; seating: 100 N. Summary measure: As specified in rows.

Group / condition S1 ID 2	Primary material	Secondary material	Taper or geometry	Group n / unit	Initial or principal force	Final/post-aging force	Absolute or percentage change
A	Zirconia	Zirconia; intermediate electroformed gold	1° (Methods p. 2)	3 systems	NR	NR	NR
B	Zirconia	Zirconia; intermediate PMMA	1° (Methods p. 2)	3 systems	NR	NR	NR
C	Zirconia	Reported Cr-Co alloy; intermediate electroformed gold	1° (Methods p. 2)	3 systems	NR	NR	NR
D	Zirconia	Reported Cr-Co alloy; intermediate PMMA	1° (Methods p. 2)	3 systems	NR	NR	NR
E	Zirconia	Acrylic; intermediate electroformed gold	1° (Methods p. 2)	3 systems	NR	NR	NR
K	Gold alloy	Reported Cr-Co alloy; intermediate electroformed gold	1° (Methods p. 2)	3 systems	NR	NR	NR

Principal comparison: Regression-line comparisons: A versus B and C versus D not significant; other slope comparisons differed.

Qualification: Absolute force endpoints appear only in plots and were not estimated. Tables 2–5 use standardized values and must not be transcribed as N. One control lost retention at 16,800 cycles; E1 stopped at 10,000 because its holder post fractured. Source calls the external alloy Cr-Co but names WIRON 88; alloy identity requires author clarification. Numeric withdrawal velocity NR; dry testing explicitly stated in Discussion.

S1 ID 3: Schwindling et al. (2017) [47]

Testing: insertion/removal cycles: Three repetitions per fitting-force level; no long-term aging; chewing/mechanical loading: NR; thermocycling: NR.

Conditions: Dry and underwater without stops; dry with stops; withdrawal/crosshead speed: 1 mm min−1; seating: 12.5, 25, 50, 75 and 100 N held 1 s; stops placed at 30 N. Summary measure: As specified in rows.

Group / condition S1 ID 3	Primary material	Secondary material	Taper or geometry	Group n / unit	Initial or principal force	Final/post-aging force	Absolute or percentage change
No stop	Zirconia	Zirconia	3°; measured mean 3.25°	8	Force NR; L/F = 0.308 (SD 0.04)	NR	NR
No stop	Zirconia	Zirconia	4°; measured mean 4.30°	8	Force NR; L/F = 0.208 (SD 0.068)	NR	NR

Group / condition S1 ID 3	Primary material	Secondary material	Taper or geometry	Group n / unit	Initial or principal force	Final/post-aging force	Absolute or percentage change
Zirconia stop	Zirconia	Zirconia	3°	8; repeated specimens	12.1 N (SD 4.5; min 4.7, max 18.0), principal maximum	NR	NR
Composite stop	Zirconia	Zirconia	3°	8; repeated specimens	12.8 N (SD 3.3; min 8.8, max 17.9), principal maximum	NR	NR

Principal comparison: L/F not significantly affected by fitting force for 2°–4° ($p \geq 0.205$); 1° data reused from earlier report.

Qualification: The 3° specimens received zirconia and then composite stops. These limit excessive loosening force. Separate force endpoints at each fitting force are graphical; NR.

S1 ID 4: Tasaka et al. (2022) [13]

Testing: insertion/removal cycles: 10,000; 30 mm/s; spring-damper fitting peak 53 N, static 39 N; chewing/mechanical loading: NR; thermocycling: NR.

Conditions: Dry force measurement; distilled-water wetting during cycling; prior water storage 37 ± 1°C for 1 week; withdrawal/crosshead speed: 1 mm/min; seating: 12.5–100 N; principal results at 100 N; composite stops bonded at 30 N. Summary measure: Mean ± SD.

Group / condition S1 ID 4	Primary material	Secondary material	Taper or geometry	Group n / unit	Initial or principal force	Final/post-aging force	Absolute or percentage change
Diamond-paste polish, no stop	Zirconia	FRC	3°	16	45.17 ± 9.73 N (F = 100 N)	NR	NR
Silicone-point polish, no stop	Zirconia	FRC	3°	16	40.10 ± 11.94 N (F = 100 N)	NR	NR
Diamond-paste polish, composite stop	Zirconia	FRC	3°	16; same specimens	19.31 ± 7.77 N	19.90 ± 9.21 N	Reported ΔL = 0.59 ± 4.55 N
Silicone-point polish, composite stop	Zirconia	FRC	3°	16; same specimens	16.12 ± 5.92 N	21.61 ± 8.82 N	Reported ΔL = 6.49 ± 8.97 N; inconsistent with endpoint means

Principal comparison: Stop-related reduction $p < 0.01$ in both groups; aging $p = 0.756$ (diamond paste), $p = 0.011$ (silicone points).

Qualification: Source reports Group 2 ΔL = 6.49 N although endpoint means differ by 5.49 N. Both source entries retained and inconsistency flagged; no correction guessed. Eight tooth forms, two crowns per form/group; no clinical outcomes.

S1 ID 10: Schwindling et al. (2016) [25]

Testing: insertion/removal cycles: 50,000 fitting/loosening cycles; 30 mm s−1; peak 53 N (static 39 N); chewing/mechanical loading: NR; thermocycling: NR.

Conditions: Dry and wet retention measurements; distilled water during cycling; withdrawal/crosshead speed: 1 mm min−1; seating: 12.5, 25, 50, 75 and 100 N held 1 s; three repetitions. Summary measure: As specified in rows.

Group / condition S1 ID 10	Primary material	Secondary material	Taper or geometry	Group n / unit	Initial or principal force	Final/post-aging force	Absolute or percentage change
1°	Zirconia	Zirconia	1°; actual mean 1.31°	8	Force NR; L/F = 0.632 (SD 0.038)	NR	NR
2°	Zirconia	Zirconia	2°; actual mean 2.26°	8	Force NR; L/F = 0.526 (SD 0.022)	NR	NR

Principal comparison: Aging-related change in L/F: 0.026 (SD 0.103), $p > 0.28$ for paired tests; wet versus dry ΔL/F = 0.041 (SD 0.045), $p = 0.025$.

Qualification: Absolute force endpoints are graphical; values were not estimated or reconstructed by multiplying L/F by fitting load. Results report dimensionless force ratios, not forces in N.

S1 ID 11: Merk et al. (2016) [24]

Testing: insertion/removal cycles: 20 pull-off tests per assembly; chewing/mechanical loading: NR; thermocycling: NR.

Conditions: Artificial saliva (Glandosane); withdrawal/crosshead speed: 50 mm/min; seating: 5 kg for 20 s before each pull-off. Summary measure: Mean ± SD; principal summary across 20 pull-offs, not a separately reported initial or final aging endpoint.

Group / condition S1 ID 11	Primary material	Secondary material	Taper or geometry	Group n / unit	Initial or principal force	Final/post-aging force	Absolute or percentage change
CoCr/Y-TZP	CoCr	Y-TZP	0°	10 assemblies	17.38 ± 6.98 N (principal)	NR	NR
Y-TZP/Y-TZP	Y-TZP	Y-TZP	0°	10 assemblies	17.63 ± 5.16 N (principal)	NR	NR
CoCr/Electroformed gold	CoCr	Electroformed gold	0°	10 assemblies	10.38 ± 2.85 N (principal)	NR	NR
Y-TZP/Electroformed gold	Y-TZP	Electroformed gold	0°	10 assemblies	7.73 ± 5.37 N (principal)	NR	NR
CoCr/Y-TZP	CoCr	Y-TZP	1°	10 assemblies	26.44 ± 5.48 N (principal)	NR	NR
Y-TZP/Y-TZP	Y-TZP	Y-TZP	1°	10 assemblies	17.92 ± 6.92 N (principal)	NR	NR
CoCr/Electroformed gold	CoCr	Electroformed gold	1°	10 assemblies	22.40 ± 8.73 N (principal)	NR	NR
Y-TZP/Electroformed gold	Y-TZP	Electroformed gold	1°	10 assemblies	14.63 ± 8.26 N (principal)	NR	NR
CoCr/Y-TZP	CoCr	Y-TZP	2°	10 assemblies	16.86 ± 8.59 N (principal)	NR	NR

Group / condition S1 ID 11	Primary material	Secondary material	Taper or geometry	Group n / unit	Initial or principal force	Final/post-aging force	Absolute or percentage change
Y-TZP/Y-TZP	Y-TZP	Y-TZP	2°	10 assemblies	22.71 ± 7.31 N (principal)	NR	NR
CoCr/Electroformed gold	CoCr	Electroformed gold	2°	10 assemblies	14.74 ± 6.05 N (principal)	NR	NR
Y-TZP/Electroformed gold	Y-TZP	Electroformed gold	2°	10 assemblies	11.35 ± 4.87 N (principal)	NR	NR

Principal comparison: One-/two-way ANOVA and Scheffé comparisons. Taper effect: C/Z $p = 0.009$, C/G $p = 0.001$; Z/Z $p = 0.167$, Z/G $p = 0.069$.

Qualification: Sixty primary crowns were combined with two secondary types, yielding 120 assemblies. n = 10 per material-pair/taper group; 20 measurements are repeated tests, not 20 independent specimens. Gold secondaries were reinforced by CoCr tertiary structures. 0° chamfer differs from the tangential ending at 1°/2°.

S1 ID 12: Merk et al. (2016) [7]

Testing: insertion/removal cycles: 20 pull-off tests per assembly; chewing/mechanical loading: NR; thermocycling: NR.

Conditions: Artificial saliva (Glandosane); withdrawal/crosshead speed: 50 mm/min; seating: 5 kg for 20 s. Summary measure: Mean ± SD; principal summary across 20 pull-offs, not a separately reported initial or final aging endpoint.

Group / condition S1 ID 12	Primary material	Secondary material	Taper or geometry	Group n / unit	Initial or principal force	Final/post-aging force	Absolute or percentage change
Milled PEEK	Zirconia	Milled PEEK	0°	10	13.83 ± 7.82 N (principal)	NR	NR
Pellet-pressed PEEK	Zirconia	Pellet-pressed PEEK	0°	10	22.83 ± 5.94 N (principal)	NR	NR
Granule-pressed PEEK	Zirconia	Granule-pressed PEEK	0°	10	15.87 ± 2.58 N (principal)	NR	NR
Milled PEEK	Zirconia	Milled PEEK	1°	10	6.07 ± 3.01 N (principal)	NR	NR
Pellet-pressed PEEK	Zirconia	Pellet-pressed PEEK	1°	10	21.06 ± 8.60 N (principal)	NR	NR
Granule-pressed PEEK	Zirconia	Granule-pressed PEEK	1°	10	27.00 ± 10.05 N (principal)	NR	NR
Milled PEEK	Zirconia	Milled PEEK	2°	10	14.10 ± 8.19 N (principal)	NR	NR
Pellet-pressed PEEK	Zirconia	Pellet-pressed PEEK	2°	10	19.84 ± 7.13 N (principal)	NR	NR
Granule-pressed PEEK	Zirconia	Granule-pressed PEEK	2°	10	19.05 ± 8.25 N (principal)	NR	NR

Principal comparison: Two-way interaction of taper and fabrication p = 0.001; cycling effect p = 0.354. Within 0°, pellet-pressed exceeded the other methods (p = 0.005); within 1°, milled was lower than pressed (p < 0.001).

Qualification: Ninety PEEK secondary crowns; n = 10 per fabrication/taper group. Repeated pull-offs are not independent samples; no long-term aging endpoints.

S1 ID 13: Luft et al. (2021) [23]

Testing: insertion/removal cycles: 10,000 joining/separation cycles; chewing/mechanical loading: NR; thermocycling: NR.

Conditions: Continuous artificial saliva (Glandosane); withdrawal/crosshead speed: 120 mm/min; seating: 50 N. Summary measure: As specified in rows.

Group / condition S1 ID 13	Primary material	Secondary material	Taper or geometry	Group n / unit	Initial or principal force	Final/post-aging force	Absolute or percentage change
GG	Cast gold alloy	Cast gold alloy	0°	10	NR	NR	−1.58% (reported)
NN	Cast CoCr	Cast CoCr	0°	10	NR	NR	−36.17% (reported)
ZG	Y-TZP	Electroformed gold	0°	10	8.3 N (mean; SD NR)	NR	NR
CC NN	Milled CoCr	Milled CoCr	0°	10	NR	NR	−10.82% (reported)
CC ZN	Y-TZP	Milled CoCr	0°	10	NR	NR	−6.33% (reported)
CC ZP	Y-TZP	PEEK	0°	10	1.36 N (mean; SD NR)	NR	NR

Principal comparison: Baseline-to-final loss significant for ZG (p = 0.010) and CC ZP (p = 0.049).

Qualification: Most endpoint values and SDs appear only in Fig. 4. Two initial means and four percentage losses are explicitly stated in the Results. Gold secondaries had CoCr reinforcement.

S1 ID 14: Igarashi et al. (2023) [27]

Testing: insertion/removal cycles: 20,000; 1 Hz; first 100 cycles omitted for stabilization; chewing/mechanical loading: NR; thermocycling: NR.

Conditions: 5 mL artificial saliva (Saliveht); withdrawal/crosshead speed: NR; cycle frequency 1 Hz; seating: 50 Ncm for 1 s, as reported. Summary measure: As specified in rows.

Group / condition S1 ID 14	Primary material	Secondary material	Taper or geometry	Group n / unit	Initial or principal force	Final/post-aging force	Absolute or percentage change
Test 1	Zirconia	PEKK	2°; buccal/lingual friction heights 5.0/3.0 mm	6	NR	NR	−11.20 Ncm (95% CI −21.52 to −0.87); p = 0.035
Test 2	Titanium	PEKK	2°; buccal/lingual friction heights 5.0/3.0 mm	6	NR	NR	−17.79 Ncm (95% CI −28.12 to −7.47); p = 0.001
Test 3	CoCr	PEKK	2°; buccal/lingual friction heights 5.0/3.0 mm	6	NR	NR	+0.28 Ncm (95% CI −10.04 to 10.61); p = 0.956
Test 4	PEKK	PEKK	2°; buccal/lingual friction heights 5.0/3.0 mm	6	NR	NR	+19.21 Ncm (95% CI 8.89 to 29.53); p = 0.001

Group / condition S1 ID 14	Primary material	Secondary material	Taper or geometry	Group n / unit	Initial or principal force	Final/post-aging force	Absolute or percentage change
Test 5	Gold	PEKK	2°; buccal/lingual friction heights 5.0/3.0 mm	6	NR	NR	+14.41 Ncm (95% CI 4.09 to 24.73); p = 0.008
Control	Gold	Galvano-gold	2°; buccal/lingual friction heights 5.0/3.0 mm	6	NR	NR	+10.72 Ncm (95% CI 0.40 to 21.04); p = 0.042

Principal comparison: Two-way repeated-measures ANOVA; Benjamini–Hochberg corrected comparisons. Rows give 20,000-cycle versus baseline differences.

Qualification: Units are internally inconsistent: Methods labels retention and seating force Ncm, while Fig. 4 labels its force axis N; Table 1 itself gives no unit. Ncm is retained from Methods without conversion, and the discrepancy requires source-author clarification. Baseline/final forces are graphical. CoCr tertiary caps reinforced secondaries; 25 µm CAD space for PEKK.

S1 ID 15: Priester et al. (2021) [33]

Testing: insertion/removal cycles: 10 tests at each of 1, 2, 5, 10 mm/min; long-term: one 3-crown block each of PEEK/PEEK and zirconia/PEEK for 10,000 cycles; chewing/mechanical loading: NR; thermocycling: NR.

Conditions: NR; withdrawal/crosshead speed: 1, 2, 5, 10 mm/min; long-term 10 mm/min; seating: Manual initial assembly; numeric fitting load NR. Summary measure: As specified in rows.

Group / condition S1 ID 15	Primary material	Secondary material	Taper or geometry	Group n / unit	Initial or principal force	Final/post-aging force	Absolute or percentage change
CoCr/3C	CoCr	PEEK	0°	3 blocks of 3 crowns	10.63 (0.531) N (mean (SD); principal)	NR	NR
CoCr/2C	CoCr	PEEK	0°	3 blocks of 2 crowns	5.713 ± 1.223 N (mean (SD); principal)	NR	NR
CoCr/1C	CoCr	PEEK	0°	9 single crowns	4.918 (0.257) N (mean (SD); principal)	NR	NR
PEEK/3C	PEEK	PEEK	0°	3 blocks of 3 crowns	19.12 (0.533) N (mean (SD); principal)	NR	NR
PEEK/2C	PEEK	PEEK	0°	3 blocks of 2 crowns	17.78 (2.739) N (mean (SD); principal)	NR	NR
PEEK/1C	PEEK	PEEK	0°	9 single crowns	9.19 (0.798) N (mean (SD); principal)	NR	NR
Zirconia/3C	Zirconia	PEEK	0°	3 blocks of 3 crowns	17.49 (1.46) N (mean (SD); principal)	NR	NR
Zirconia/2C	Zirconia	PEEK	0°	3 blocks of 2 crowns	5.79 (0.78) N (mean (SD); principal)	NR	NR

Group / condition S1 ID 15	Primary material	Secondary material	Taper or geometry	Group n / unit	Initial or principal force	Final/post-aging force	Absolute or percentage change
Zirconia/1C	Zirconia	PEEK	0°	9 single crowns	3.30 (1.91) N (mean (SD); principal)	NR	NR

Principal comparison: Kruskal–Wallis with Holm–Sidak comparisons; material and crown-block size affected Fmax.

Qualification: Table 2 summaries span the tested speed conditions; 3C/2C/1C are sequential subdivisions of the same crowns, not independent cohorts. Numeric long-term endpoints NR. The isolated Table 2 entry 5.713 ± 1.223 is preserved as printed.

S1 ID 16: Stock et al. (2016) [28]

Testing: insertion/removal cycles: 20 pull-off tests per assembly; chewing/mechanical loading: NR; thermocycling: NR.

Conditions: Artificial saliva (Glandosane); withdrawal/crosshead speed: 50 mm/min; seating: 50 N for 20 s. Summary measure: Mean ± SD; principal summary across 20 pull-offs, not a separately reported initial or final aging endpoint.

Group / condition S1 ID 16	Primary material	Secondary material	Taper or geometry	Group n / unit	Initial or principal force	Final/post-aging force	Absolute or percentage change
CoCr secondary	PEEK	CoCr	0°	10	15.00 ± 11.16 N (principal)	NR	NR
Zirconia secondary	PEEK	Zirconia	0°	10	16.90 ± 4.15 N (principal)	NR	NR
Electroformed gold secondary	PEEK	Electroformed gold	0°	10	26.10 ± 15.14 N (principal)	NR	NR
CoCr secondary	PEEK	CoCr	1°	10	21.40 ± 8.11 N (principal)	NR	NR
Zirconia secondary	PEEK	Zirconia	1°	10	22.80 ± 7.15 N (principal)	NR	NR
Electroformed gold secondary	PEEK	Electroformed gold	1°	10	9.60 ± 9.08 N (principal)	NR	NR
CoCr secondary	PEEK	CoCr	2°	10	31.10 ± 11.27 N (principal)	NR	NR
Zirconia secondary	PEEK	Zirconia	2°	10	38.20 ± 2.39 N (principal)	NR	NR
Electroformed gold secondary	PEEK	Electroformed gold	2°	10	14.80 ± 8.00 N (principal)	NR	NR

Principal comparison: Taper × secondary-material interaction $p < 0.001$. At 0° no material difference ($p = 0.075$); at 1° and 2° gold secondaries were lower. Significant 20-cycle decrease only for 1° CoCr ($p = 0.007$).

Qualification: Ninety assemblies across nine groups, n = 10 per group. The tabulated means summarize repeated pull-offs. PEEK primary crowns are paired with three secondary types; gold copings have CoCr tertiary reinforcement.

S1 ID 17: Stock et al. (2016) [29]

Testing: insertion/removal cycles: 20 pull-off tests per assembly; chewing/mechanical loading: NR; thermocycling: NR.

Conditions: Artificial saliva (Glandosane); withdrawal/crosshead speed: 50 mm/min; seating: 5 kg for 20 s. Summary measure: Mean ± SD; principal summary across 20 pull-offs, not a separately reported initial or final aging endpoint.

Group / condition S1 ID 17	Primary material	Secondary material	Taper or geometry	Group n / unit	Initial or principal force	Final/post-aging force	Absolute or percentage change
Milled BioHPP PEEK	PEEK	Milled BioHPP PEEK	0°	10	4.29 ± 1.48 N (principal)	NR	NR
Pellet-pressed BioHPP PEEK	PEEK	Pellet-pressed BioHPP PEEK	0°	10	14.9 ± 7.62 N (principal)	NR	NR
Granule-pressed BioHPP PEEK	PEEK	Granule-pressed BioHPP PEEK	0°	10	11.64 ± 5.74 N (principal)	NR	NR
Milled BioHPP PEEK	PEEK	Milled BioHPP PEEK	1°	10	21.12 ± 9.17 N (principal)	NR	NR
Pellet-pressed BioHPP PEEK	PEEK	Pellet-pressed BioHPP PEEK	1°	10	17.46 ± 7.13 N (principal)	NR	NR
Granule-pressed BioHPP PEEK	PEEK	Granule-pressed BioHPP PEEK	1°	10	15.11 ± 8.05 N (principal)	NR	NR
Milled BioHPP PEEK	PEEK	Milled BioHPP PEEK	2°	10	29.06 ± 9.37 N (principal)	NR	NR
Pellet-pressed BioHPP PEEK	PEEK	Pellet-pressed BioHPP PEEK	2°	10	19.73 ± 4.21 N (principal)	NR	NR
Granule-pressed BioHPP PEEK	PEEK	Granule-pressed BioHPP PEEK	2°	10	17.08 ± 9.29 N (principal)	NR	NR

Principal comparison: Taper × fabrication interaction $p < 0.001$. Significant cycling decreases: 0° pellet-pressed $p = 0.004$; 0° granule-pressed $p = 0.02$; 1° granule-pressed $p = 0.042$.

Qualification: Ninety crown assemblies; n = 10 per fabrication/taper group. Implant abutment is a standardized substrate for crown-pair testing. Twenty repeated pull-offs are not independent specimens.

S1 ID 18: Nakamura et al. (2021) [31]

Testing: insertion/removal cycles: 10,000; 950 mm/min; retention measured every 1,000 cycles; chewing/mechanical loading: NR; thermocycling: NR.

Conditions: 37°C distilled water during cycling; pull-off lubrication NR; withdrawal/crosshead speed: 50 mm/min; seating: 49 N for 20 s; average of 10 pull-offs per measurement. Summary measure: Mean ± SD.

Group / condition S1 ID 18	Primary material	Secondary material	Taper or geometry	Group n / unit	Initial or principal force	Final/post-aging force	Absolute or percentage change
PEEK	PEEK	PEEK	6°; height 7.5 mm, cervical diameter 8.4 mm	7	12.9 ± 4.0 N	6.5 ± 2.7 N	−6.4 N; −49.6% (calculated)
Gold alloy control	Type 4 gold alloy	Type 4 gold alloy	6°; height 7 mm, diameter 7 mm	7	12.3 ± 2.2 N	9.6 ± 1.8 N	−2.7 N; −22.0% (calculated)

Principal comparison: PEEK decline significant at 3,000 cycles (7.3 ± 2.0 N); final PEEK versus gold $p < 0.05$.

Qualification: Material groups differ in geometry, wall thickness and fabrication; this is not an isolated material comparison. Calculated changes use published rounded means.

S1 ID 19: Schimmel et al. (2021) [34]

Testing: insertion/removal cycles: 10,000; baseline 10 cycles; chewing/mechanical loading: NR; thermocycling: NR.

Conditions: Distilled water at 37°C; withdrawal/crosshead speed: 0.5 mm/s withdrawal; 1 mm/s insertion; seating: 40 N held 1 s. Summary measure: As specified in rows.

Group / condition S1 ID 19	Primary material	Secondary material	Taper or geometry	Group n / unit	Initial or principal force	Final/post-aging force	Absolute or percentage change
Test 1	Zirconia	PEEK	1°; retention heights 4.5 mm interdentally, 2 mm buccal/oral	10	NR	NR	NR
Test 2	Titanium	PEEK	1°; retention heights 4.5 mm interdentally, 2 mm buccal/oral	10	NR	NR	NR
Test 3	PEEK	PEEK	1°; retention heights 4.5 mm interdentally, 2 mm buccal/oral	10	NR	NR	NR
Test 4	CoCr	PEEK	1°; retention heights 4.5 mm interdentally, 2 mm buccal/oral	10	NR	NR	NR

Group / condition S1 ID 19	Primary material	Secondary material	Taper or geometry	Group n / unit	Initial or principal force	Final/post-aging force	Absolute or percentage change
Test 5	CoCr	CoCr	1°; retention heights 4.5 mm interdentally, 2 mm buccal/oral	10	NR	NR	NR
Control	Precious alloy	Precious alloy	1°; retention heights 4.5 mm interdentally, 2 mm buccal/oral	10	NR	NR	NR

Principal comparison: Zirconia/PEEK and PEEK/PEEK had significant increases at 10,000 cycles; other groups had no significant baseline-to-final change.

Qualification: Median/IQR force data are graphical; no numeric force endpoints extracted. Crown walls 0.3 mm; all secondaries completely covered by a metal tertiary structure. Original Table 4 supplies between-group p values, not endpoint force data.

S1 ID 20: Micovic Soldatovic et al. (2023) [43]

Testing: insertion/removal cycles: 10,000 insertion/removal cycles; measurements at baseline, 500, 5,000 and 10,000; chewing/mechanical loading: 50 N axial load during cyclic insertion/removal; thermocycling: 5–55°C, dwell 60 s, during thermomechanical cycling; independent thermal cycle count NR.

Conditions: Glandosane during pull-off; distilled water during aging; withdrawal/crosshead speed: 50 mm/min; five pull-offs per time point; seating: NR. Summary measure: Mean ± SD.

Group / condition S1 ID 20	Primary material	Secondary material	Taper or geometry	Group n / unit	Initial or principal force	Final/post-aging force	Absolute or percentage change
CoCr/PEEK	CoCr	PEEK	0° (parallel primary crowns)	15	13.1 ± 2.9 N	20.3 ± 4.6 N	+7.2 N (calculated)
CoCr/PEKK	CoCr	PEKK	0° (parallel primary crowns)	15	11.6 ± 2.5 N	19.6 ± 5.5 N	+8.0 N (calculated)
CoCr/Zirconia	CoCr	Zirconia	0° (parallel primary crowns)	15	10.4 ± 2.8 N	2.2 ± 1.9 N	-8.2 N (calculated)
CoCr/CoCr	CoCr	CoCr	0° (parallel primary crowns)	15	12.2 ± 2.3 N	9.1 ± 4.7 N	-3.1 N (calculated)
CoCr/PEEK 0.5 mm + CoCr tertiary	CoCr	PEEK 0.5 mm + CoCr tertiary	0° (parallel primary crowns)	15	13.1 ± 2.1 N	15.8 ± 5.6 N	+2.7 N (calculated)
CoCr/PEKK 0.5 mm + CoCr tertiary	CoCr	PEKK 0.5 mm + CoCr tertiary	0° (parallel primary crowns)	15	13.3 ± 2.0 N	15.9 ± 3.9 N	+2.6 N (calculated)
Zirconia/PEEK	Zirconia	PEEK	0° (parallel primary crowns)	15	9.1 ± 2.2 N	11.1 ± 3.8 N	+2.0 N (calculated)

Group / condition S1 ID 20	Primary material	Secondary material	Taper or geometry	Group n / unit	Initial or principal force	Final/post-aging force	Absolute or percentage change
Zirconia/PEKK	Zirconia	PEKK	0° (parallel primary crowns)	15	12.1 ± 2.6 N	18.1 ± 7.8 N	+6.0 N (calculated)
Zirconia/Zirconia	Zirconia	Zirconia	0° (parallel primary crowns)	15	9.8 ± 2.3 N	1.6 ± 1.3 N	-8.2 N (calculated)
Zirconia/CoCr	Zirconia	CoCr	0° (parallel primary crowns)	15	10.9 ± 2.0 N	6.1 ± 5.0 N	-4.8 N (calculated)
Zirconia/PEEK 0.5 mm + CoCr tertiary	Zirconia	PEEK 0.5 mm + CoCr tertiary	0° (parallel primary crowns)	15	13.0 ± 2.3 N	16.1 ± 6.4 N	+3.1 N (calculated)
Zirconia/PEKK 0.5 mm + CoCr tertiary	Zirconia	PEKK 0.5 mm + CoCr tertiary	0° (parallel primary crowns)	15	13.7 ± 1.5 N	18.5 ± 4.3 N	+4.8 N (calculated)

Principal comparison: Material pairing and cycle count $p < 0.001$; polymer secondary groups increased; zirconia/zirconia and zirconia/CoCr decreased.

Qualification: Secondary crowns were individually adjusted toward a 10–15 N target before aging. 0.5 mm polymer copings have a CoCr tertiary crown; full-contour polymer groups do not. Calculated changes use rounded means.

S1 ID 21: Awad et al. (2025) [22]

Testing: insertion/removal cycles: 1,440 insertion/removal cycles; chewing/mechanical loading: 240,000 biaxial cycles; 49 N, 1.6 Hz; 3 mm vertical/0.7 mm horizontal path, 60 mm/s; thermocycling: NR.

Conditions: Artificial saliva (Glandosane); room temperature; withdrawal/crosshead speed: 50 mm/min; seating: 50 N for 20 s; 10 baseline and three final measurements. Summary measure: Mean ± SD.

Group / condition S1 ID 21	Primary material	Secondary material	Taper or geometry	Group n / unit	Initial or principal force	Final/post-aging force	Absolute or percentage change
Zirconia	Zirconia	Zirconia	NR	6 prostheses	5.5982 ± 0.04987 N	4.3277 ± 0.26277 N	-1.271 N; -22.7% (calculated from reported means)
PEKK	PEKK	PEKK	NR	6 prostheses	10.2412 ± 0.71109 N	6.9064 ± 1.07965 N	-3.335 N; -32.6% (calculated from reported means)
CoCr	CoCr	CoCr	NR	6 prostheses	9.5205 ± 1.05162 N	5.8143 ± 0.85251 N	-3.706 N; -38.9% (calculated from reported means)

Principal comparison: Within-group decreases $p = 0.0001$; final PEKK versus CoCr $p = 0.015$.

Qualification: Whole-prosthesis outcome from combined support: two canine teeth plus two molar implants. Cannot be generalized to tooth-only support. Thermocycling NR.

S1 ID 22: Mostafa et al. (2021) [30]

Testing: insertion/removal cycles: Initial pull-off; aging NR; chewing/mechanical loading: NR; thermocycling: NR.

Conditions: NR; withdrawal/crosshead speed: 50 mm/min (stated in Discussion); seating: 50 N for 20 s. Summary measure: Mean ± SD.

Group / condition S1 ID 22	Primary material	Secondary material	Taper or geometry	Group n / unit	Initial or principal force	Final/post-aging force	Absolute or percentage change
A	PEKK (Pekkton)	PEKK	Prepared abutments 3°; crown taper NR	6 frameworks; 24 primary crowns	10.40 ± 1.49 N	NR	NR
B	Zirconia	PEKK	Prepared abutments 3°; crown taper NR	6 frameworks; 24 primary crowns	6.78 ± 1.05 N	NR	NR

Principal comparison: Student t-test $p = 0.001$.

Qualification: Kennedy Class I tooth-supported model; dislodging force is measured for the four-crown framework. Loading conditions or long-term retention cannot be inferred from this initial comparison.

S1 ID 27: Donia et al. (2022) [26]

Testing: insertion/removal cycles: 1,440; 50 mm/min; interim 360, 720 and 1,080; chewing/mechanical loading: NR; thermocycling: NR.

Conditions: NR; withdrawal/crosshead speed: 5 mm/min pull-off; seating: NR. Summary measure: Mean ± SD.

Group / condition S1 ID 27	Primary material	Secondary material	Taper or geometry	Group n / unit	Initial or principal force	Final/post-aging force	Absolute or percentage change
1	PEEK	PEEK	2°; secondary 1 mm	5	22.62 ± 3.87 N	9.39 ± 0.72 N	-13.23 N; -58.5% (calculated from reported means)
2	PEEK	PEEK	6°; secondary 1 mm	5	19.61 ± 0.87 N	8.94 ± 1.33 N	-10.67 N; -54.4% (calculated from reported means)
3	PEEK	PEEK	2°; secondary 2 mm	5	24.88 ± 4.93 N	11.16 ± 0.71 N	-13.72 N; -55.1% (calculated from reported means)
4	PEEK	PEEK	6°; secondary 2 mm	5	19.37 ± 2.31 N	8.53 ± 0.88 N	-10.84 N; -56.0% (calculated from reported means)

Principal comparison: Between-group final $p = 0.002$; within-group changes $p \leq 0.004$ (Table 2).

Qualification: Single crown-pair engineering tests on implant abutments; crown height 6 mm. The abstract incorrectly refers to electroformed secondaries and stable retention; Table 2 shows declines for all PEEK/PEEK groups. No gold-secondary group was tested. Lubrication and seating load NR.

S1 ID 28: Yoshikawa et al. (2019) [37]

Testing: insertion/removal cycles: 10,000; approximately 0.5 Hz; 4 mm amplitude; 50 N cyclic load; chewing/mechanical loading: NR; thermocycling: NR.

Conditions: NR; withdrawal/crosshead speed: 40 mm/min; seating: 50 or 100 N for 5 s; five measurements averaged. Summary measure: As specified in rows.

Group / condition S1 ID 28	Primary material	Secondary material	Taper or geometry	Group n / unit	Initial or principal force	Final/post-aging force	Absolute or percentage change
50 N seating	Ce-TZP/Al2O3	Ce-TZP/Al2O3	2°	5	20.8 N (mean; SD NR)	18.5 N (mean; SD NR)	−11% (reported)

Group / condition S1 ID 28	Primary material	Secondary material	Taper or geometry	Group n / unit	Initial or principal force	Final/post-aging force	Absolute or percentage change
50 N seating	Ce-TZP/Al2O3	Ce-TZP/Al2O3	4°	5	8.5 N (mean; SD NR)	6.0 N (mean; SD NR)	−30% (reported)
100 N seating	Ce-TZP/Al2O3	Ce-TZP/Al2O3	2°	5	40.7 N (mean; SD NR)	36.5 N (mean; SD NR)	−10% (reported)
100 N seating	Ce-TZP/Al2O3	Ce-TZP/Al2O3	4°	5	17.1 N (mean; SD NR)	12.7 N (mean; SD NR)	−26% (reported)

Principal comparison: Taper and cycling significant; at 50 N, 2° changed after 10,000 cycles and 4° after each assessed interval.

Qualification: Same crown specimens evaluated at both seating loads. Force means explicitly reported in Results; SDs shown graphically were not estimated. Secondary wall 0.4 mm, CAD gap 10 µm. Lubrication NR.

S1 ID 29: Fischer et al. (2020) [8]

Testing: insertion/removal cycles: 360; first-cycle baseline; 30/90/180/270 interim sets; chewing/mechanical loading: NR; thermocycling: NR.

Conditions: Dry; withdrawal/crosshead speed: Linear speed NR; 18 cycles/min and 2 mm displacement; seating: NR. Summary measure: Mean ± SD.

Group / condition S1 ID 29	Primary material	Secondary material	Taper or geometry	Group n / unit	Initial or principal force	Final/post-aging force	Absolute or percentage change
13-CC-0°	CoCr	CoCr	Canine 13; 0°	15/group; three assigned per time interval	1.90 ± 0.51 N	1.38 ± 0.19 N	NR
13-CC-2°	CoCr	CoCr	Canine 13; 2°	15/group; three assigned per time interval	1.60 ± 0.36 N	1.51 ± 0.71 N	NR
13-CZ-0°	Zirconia	CoCr	Canine 13; 0°	15/group; three assigned per time interval	5.92 ± 0.35 N	3.71 ± 0.58 N	NR
13-CZ-2°	Zirconia	CoCr	Canine 13; 2°	15/group; three assigned per time interval	4.52 ± 0.62 N	2.10 ± 0.61 N	NR
26-CC-0°	CoCr	CoCr	Molar 26; 0°	15/group; three assigned per time interval	2.73 ± 0.67 N	5.26 ± 0.62 N	NR
26-CC-2°	CoCr	CoCr	Molar 26; 2°	15/group; three assigned per time interval	1.40 ± 0.42 N	2.42 ± 0.22 N	NR
26-CZ-0°	Zirconia	CoCr	Molar 26; 0°	15/group; three assigned per time interval	3.82 ± 0.38 N	5.98 ± 0.45 N	NR

Group / condition S1 ID 29	Primary material	Secondary material	Taper or geometry	Group n / unit	Initial or principal force	Final/post-aging force	Absolute or percentage change
26-CZ-2°	Zirconia	CoCr	Molar 26; 2°	15/group; three assigned per time interval	3.01 ± 0.22 N	3.51 ± 0.39 N	NR

Principal comparison: Descriptive comparisons; inferential retention comparison NR.

Qualification: The paper reports 15 specimens/group and three per time interval. Endpoint contrasts need not be paired specimen changes; changes are not calculated. CZ denotes zirconia primary with CoCr secondary, not zirconia/zirconia.

S1 ID 30: Kawilarang et al. (2020) [36]

Testing: insertion/removal cycles: NR; pull-off comparison; chewing/mechanical loading: NR; thermocycling: NR.

Conditions: Artificial saliva; withdrawal/crosshead speed: 50 mm/minute; seating: NR. Summary measure: Mean ± SD.

Group / condition S1 ID 30	Primary material	Secondary material	Taper or geometry	Group n / unit	Initial or principal force	Final/post-aging force	Absolute or percentage change
CoCr/0°	CoCr	CoCr	0°	4	18.25 ± 0.83 N (principal)	NR	NR
CoCr/1°	CoCr	CoCr	1°	4	13.98 ± 0.40 N (principal)	NR	NR
CoCr/2°	CoCr	CoCr	2°	4	10.28 ± 0.51 N (principal)	NR	NR
Zirconia/0°	Zirconia	Zirconia	0°	4	22.48 ± 1.12 N (principal)	NR	NR
Zirconia/1°	Zirconia	Zirconia	1°	4	19.28 ± 0.50 N (principal)	NR	NR
Zirconia/2°	Zirconia	Zirconia	2°	4	15.35 ± 0.53 N (principal)	NR	NR

Principal comparison: Two-way ANOVA: material, angulation and interaction; LSD comparisons in Table 5.

Qualification: Small single-pair groups. Taper and material comparisons are initial mechanical findings; no cyclic aging endpoints.

S1 ID 31: Çelik Güven et al. (2017) [44]

Testing: insertion/removal cycles: 5,000; 300 mm/min, 2.2 Hz; retention every 500 cycles; chewing/mechanical loading: NR; thermocycling: NR.

Conditions: Artificial saliva prepared by Shannon procedure; withdrawal/crosshead speed: 20 mm/min withdrawal, 10 mm/min descent; seating: 50 N. Summary measure: Mean ± SD.

Group / condition S1 ID 31	Primary material	Secondary material	Taper or geometry	Group n / unit	Initial or principal force	Final/post-aging force	Absolute or percentage change
AA	Gold alloy	Gold alloy	4°	6	19.27 ± 4.91 N	32.14 ± 3.55 N	+12.87 N; +66.8% (calculated from reported means)
LL	Laser-sintered CoCr	Laser-sintered CoCr	4°	6	32.89 ± 9.82 N	32.65 ± 2.56 N	-0.24 N; -0.7% (calculated from reported means)
CC	Cast CoCr	Cast CoCr	4°	6	16.63 ± 7.75 N	29.36 ± 6.29 N	+12.73 N; +76.5% (calculated from reported means)

Group / condition S1 ID 31	Primary material	Secondary material	Taper or geometry	Group n / unit	Initial or principal force	Final/post-aging force	Absolute or percentage change
ZA	Zirconia	Electroformed gold	4°	6	11.86 ± 7.48 N	13.26 ± 6.19 N	+1.4 N; +11.8% (calculated from reported means)
TT	Titanium	Titanium	4°	6	20.8 ± 11.02 N	20.682 ± 9.16 N	-0.118 N; -0.6% (calculated from reported means)
AA	Gold alloy	Gold alloy	6°	6	14.33 ± 3.76 N	23.89 ± 6.1 N	+9.56 N; +66.7% (calculated from reported means)
LL	Laser-sintered CoCr	Laser-sintered CoCr	6°	6	10.07 ± 2.7 N	21.21 ± 5.99 N	+11.14 N; +110.6% (calculated from reported means)
CC	Cast CoCr	Cast CoCr	6°	6	8.36 ± 4.25 N	21.08 ± 3.5 N	+12.72 N; +152.2% (calculated from reported means)
ZA	Zirconia	Electroformed gold	6°	6	5.41 ± 0.66 N	6.27 ± 1.13 N	+0.86 N; +15.9% (calculated from reported means)
TT	Titanium	Titanium	6°	6	10.11 ± 4.8 N	17.62 ± 5.78 N	+7.51 N; +74.3% (calculated from reported means)

Principal comparison: Between-material comparisons p = 0.004 initially and p = 0.001 finally for each taper.

Qualification: Standardized steel dies imitate tooth/implant abutments. Single crown-pair engineering evidence. Ten initial seating/separation cycles and three force repetitions; taper convention retained.

S1 ID 32: Goguță et al. (2021) [45]

Testing: insertion/removal cycles: Limited repeated retention tests; no long-term aging endpoints; chewing/mechanical loading: NR; thermocycling: NR.

Conditions: Dry or artificial saliva, as specified by group; withdrawal/crosshead speed: 1 mm/minute; seating: NR. Summary measure: Mean (SD).

Group / condition S1 ID 32	Primary material	Secondary material	Taper or geometry	Group n / unit	Initial or principal force	Final/post-aging force	Absolute or percentage change
Zirconia canine dry	Zirconia	SLM CoCr	6°; canine	7 observations per condition	10.33 (2.90) N (principal)	NR	NR
Zirconia canine saliva	Zirconia	SLM CoCr	6°; canine	7 observations per condition	12.14 (2.61) N (principal)	NR	NR
Zirconia molar dry	Zirconia	SLM CoCr	6°; molar	7 observations per condition	4.83 (0.56) N (principal)	NR	NR
Zirconia molar saliva	Zirconia	SLM CoCr	6°; molar	7 observations per condition	8.16 (1.42) N (principal)	NR	NR

Group / condition S1 ID 32	Primary material	Secondary material	Taper or geometry	Group n / unit	Initial or principal force	Final/post-aging force	Absolute or percentage change
SLS CoCr canine dry	SLS CoCr	SLM CoCr	6°; canine	7 observations per condition	14.31 (0.73) N (principal)	NR	NR
SLS CoCr canine saliva	SLS CoCr	SLM CoCr	6°; canine	7 observations per condition	15.67 (1.13) N (principal)	NR	NR
SLS CoCr molar dry	SLS CoCr	SLM CoCr	6°; molar	7 observations per condition	11.54 (1.70) N (principal)	NR	NR
SLS CoCr molar saliva	SLS CoCr	SLM CoCr	6°; molar	7 observations per condition	14.05 (1.93) N (principal)	NR	NR
SLM CoCr canine dry	SLM CoCr	SLM CoCr	6°; canine	7 observations per condition	3.16 (0.19) N (principal)	NR	NR
SLM CoCr canine saliva	SLM CoCr	SLM CoCr	6°; canine	7 observations per condition	3.85 (0.39) N (principal)	NR	NR
SLM CoCr molar dry	SLM CoCr	SLM CoCr	6°; molar	7 observations per condition	4.52 (0.89) N (principal)	NR	NR
SLM CoCr molar saliva	SLM CoCr	SLM CoCr	6°; molar	7 observations per condition	5.59 (0.71) N (principal)	NR	NR

Principal comparison: Three-way ANOVA/Tukey: material/fabrication and tooth form affected retention; zirconia versus SLM and versus SLS $p < 0.001$.

Qualification: 84 observations across material × tooth × saliva conditions. Report uses observations/replicates and also describes 14 crowns per tooth/material; preserve this distinction. Table 1 identifies CoCr, despite occasional “stainless steel” wording in narrative.

S1 ID 33: Abdallah et al. (2023) [46]

Testing: insertion/removal cycles: 270 manual insertion/removal cycles; chewing/mechanical loading: 50,000 biaxial cycles; 50 N; thermocycling: NR.

Conditions: Artificial saliva (Glandosane); withdrawal/crosshead speed: 50 mm/min; seating: 50 N for 20 s. Summary measure: Mean ± SD.

Group / condition S1 ID 33	Primary material	Secondary material	Taper or geometry	Group n / unit	Initial or principal force	Final/post-aging force	Absolute or percentage change
I	Zirconia	CoCr	2°–4°; 1.5–2 mm parallel cervical band	7 coping sets/RPDs per group	4.746 ± 1.329 N	1.935 ± 0.268 N	-2.811 N; -59.2% (calculated from reported means)
II	CoCr	CoCr	2°–4°; 1.5–2 mm parallel cervical band	7 coping sets/RPDs per group	1.999 ± 0.238 N	0.583 ± 0.018 N	-1.416 N; -70.8% (calculated from reported means)

Principal comparison: Baseline group comparison $p = 0.0047$; final $p < 0.0001$. Within-group $p = 0.0056$ (zirconia), $p < 0.0001$ (CoCr).

Qualification: Tooth-supported Kennedy Class I framework model. Methods specify 14 sets of primary copings, seven sets per group, rather than 14 individual crowns. Force is measured for the whole RPD; two printed support models were described.

S1 ID 34: Milić-Lemić et al. (2020) [49]

Testing: insertion/removal cycles: 900; repeated manually until ≥10 consecutive stable readings; chewing/mechanical loading: NR; thermocycling: NR.

Conditions: Biotene saliva substitute with physiological saline (1:2); withdrawal/crosshead speed: NR; manual dynamometer; seating: Finger pressure. Summary measure: Mean ± SD and median/IQR, as reported.

Group / condition S1 ID 34	Primary material	Secondary material	Taper or geometry	Group n / unit	Initial or principal force	Final/post-aging force	Absolute or percentage change
PEEK	Zirconia	PEEK	NR; maxillary canine model	20	9.3 ± 1.2 N; median 9.7, IQR 2.4	4.1 ± 0.5 N; median 4.3, IQR 0.8	-5.2 N; -55.9% (calculated from reported means)
Au-ZrO2	Zirconia	Electroplated gold coping with zirconia tertiary structure	NR; maxillary canine model	20	7 ± 2.6 N; median 7.3, IQR 2.4	3 ± 0.9 N; median 3.2, IQR 0.6	-4 N; -57.1% (calculated from reported means)

Principal comparison: Baseline between groups p = 0.001; final reported p = 0.000. No 800-to-900-cycle change (p = 0.157 and 0.063).

Qualification: Final force represents initial settling, not long-term aging. Corrected original S1: the gold-coping group has a zirconia tertiary structure, not PEEK. Manual force application and a 0–20 N dynamometer limit comparisons.

S1 ID 35: Tasaka et al. (2022) [10]

Testing: insertion/removal cycles: 10,000; 30 mm/s; fitting peak 53 N (static 39 N); chewing/mechanical loading: NR; thermocycling: NR.

Conditions: Dry or thin water film for force measurement; distilled-water wetting during cycling; water storage 37°C for 1 week; withdrawal/crosshead speed: 1 mm/min; seating: Up to 100 N to reach final seating position. Summary measure: As specified in rows.

Group / condition S1 ID 35	Primary material	Secondary material	Taper or geometry	Group n / unit	Initial or principal force	Final/post-aging force	Absolute or percentage change
1 dry	Zirconia	FRC	0°; +10 µm milling offset; diamond paste	16	9.8; 2.5–14.0 N (median; range)	11.5; 1.7–21.5 N (median; range)	NR
1 wet	Zirconia	FRC	0°; +10 µm milling offset; diamond paste	16	10.0; 1.8–26.0 N (median; range)	7.4; 0.7–24.9 N (median; range)	NR
2 dry	Zirconia	FRC	0°; −10 µm offset; diamond paste	16	7.7; 4.7–10.3 N (median; range)	4.6; 0.9–10.8 N (median; range)	NR
2 wet	Zirconia	FRC	0°; −10 µm offset; diamond paste	16	9.3; 3.3–15.1 N (median; range)	3.4; 0.9–14.7 N (median; range)	NR
3 dry	Zirconia	FRC	0°; −10 µm offset; silicone points	16	9.0; 5.2–14.8 N (median; range)	2.6; 0.1–7.5 N (median; range)	NR
3 wet	Zirconia	FRC	0°; −10 µm offset; silicone points	16	15.1; 8.3–23.8 N (median; range)	2.5; 0.3–8.7 N (median; range)	NR

Principal comparison: Aging decline in groups 2 and 3 $p < 0.003$; no aging effect group 1 (dry $p = 0.363$, wet $p = 0.552$).

Qualification: Same crowns measured dry and wet. Forty-eight FRC secondaries across eight tooth forms. Post-milling retention was adjusted toward 5–15 N; outcomes depend on offset and finishing.

S1 ID 36: Kotthaus et al. (2019) [32]

Testing: insertion/removal cycles: 10,000; 120 mm/min; chewing/mechanical loading: NR; thermocycling: NR.

Conditions: Biotene diluted with distilled water (2:1); withdrawal/crosshead speed: 120 mm/min; seating: Joining movement stopped at >30 N. Summary measure: As specified in rows.

Group / condition S1 ID 36	Primary material	Secondary material	Taper or geometry	Group n / unit	Initial or principal force	Final/post-aging force	Absolute or percentage change
1	Gold alloy NEOCAST 3	PEKK	0°	10	9.0 N (mean; SD NR)	15.1 N (mean; SD NR)	+6.1 N; +67.8% (calculated from reported means)
2	Non-precious alloy Girobond NB	PEKK	0°	10	8.1 N (mean; SD NR)	20.6 N (mean; SD NR)	+12.5 N; +154.3% (calculated from reported means)
3	Zirconia Cercon base	PEKK	0°	10	11.0 N (mean; SD NR)	29.4 N (mean; SD NR)	+18.4 N; +167.3% (calculated from reported means)
4	PEKK	PEKK	0°	10	4.6 N (mean; SD NR)	14.8 N (mean; SD NR)	+10.2 N; +221.7% (calculated from reported means)

Principal comparison: Inferential between-group test NR; descriptive retention progression.

Qualification: The opening abstract mentions 5,000 simulated cycles, but Methods and Results report 10,000 completed cycles. Final means are explicitly stated. The hex screw is a specimen-mounting component, not a clinical auxiliary retainer.

S1 ID 39: Enaba (2022) [38]

Testing: insertion/removal cycles: 20 insertion/removal cycles per sample; chewing/mechanical loading: NR; thermocycling: NR.

Conditions: NR; withdrawal/crosshead speed: 50 mm/min; seating: NR. Summary measure: Mean ± SD.

Group / condition S1 ID 39	Primary material	Secondary material	Taper or geometry	Group n / unit	Initial or principal force	Final/post-aging force	Absolute or percentage change
PEEK/0°	PEEK	PEEK	0°	5	6.88 ± 0.64 N (principal)	NR	NR
PEEK/1°	PEEK	PEEK	1°	5	6.43 ± 0.75 N (principal)	NR	NR
PEEK/2°	PEEK	PEEK	2°	5	5.09 ± 0.48 N (principal)	NR	NR
Graphene-reinforced acrylic/0°	Graphene-reinforced acrylic	Graphene-reinforced acrylic	0°	5	5.01 ± 0.60 N (principal)	NR	NR
Graphene-reinforced acrylic/1°	Graphene-reinforced acrylic	Graphene-reinforced acrylic	1°	5	4.84 ± 0.44 N (principal)	NR	NR

Group / condition S1 ID 39	Primary material	Secondary material	Taper or geometry	Group n / unit	Initial or principal force	Final/post-aging force	Absolute or percentage change
Graphene-reinforced acrylic/2°	Graphene-reinforced acrylic	Graphene-reinforced acrylic	2°	5	4.14 ± 0.36 N (principal)	NR	NR

Principal comparison: PEEK versus graphene-acrylic p = 0.0001 at each taper; 2° lower than 0°/1°.

Qualification: Principal averages across 20 pull-offs, not separately reported baseline/final aging values. Primary crown and die are one milled object. Lubrication and seating load NR; no long-term aging.

S1 ID 40: Linek et al. (2016) [35]

Testing: insertion/removal cycles: 10,000 load-change cycles; 3 mm travel each direction; 0.5 s pause; chewing/mechanical loading: NR; thermocycling: NR.

Conditions: Distilled water at 36°C; withdrawal/crosshead speed: 15.0 mm/min; seating: NR. Summary measure: Median and mean ± SD.

Group / condition S1 ID 40	Primary material	Secondary material	Taper or geometry	Group n / unit	Initial or principal force	Final/post-aging force	Absolute or percentage change
A	High-gold alloy	High-gold alloy	0° cylindrical	10 assemblies	11.1 N (median); 14.9 ± 12.8 N (mean ± SD)	3.3 N (median); 4.0 ± 2.6 N (mean ± SD)	−70% (reported tendency)
B	High-gold alloy	CoCr	0° cylindrical	10 assemblies	10.7 N (median); 11.0 ± 1.9 N (mean ± SD)	5.9 N (median); 6.5 ± 2.5 N (mean ± SD)	−45% (reported tendency)
C	High-gold alloy	Electroformed gold	0° cylindrical	10 assemblies	5.6 N (median); 5.3 ± 1.8 N (mean ± SD)	NR; series stopped when forces exceeded 50 N	NR
D	Titanium	Titanium	0° cylindrical	10 assemblies	18.2 N (median); 18.6 ± 6.6 N (mean ± SD)	2.1 N (median); 2.4 ± 1.5 N (mean ± SD)	−88% (reported tendency)
E	Titanium	Electroformed gold	0° cylindrical	10 assemblies	5.9 N (median); 6.1 ± 1.0 N (mean ± SD)	4.4 N (median); 4.7 ± 1.5 N (mean ± SD)	−25% (reported tendency)
F	Lithium disilicate (IPS Empress 2)	Electroformed gold	0° cylindrical	10 assemblies	3.1 N (median); 3.4 ± 1.1 N (mean ± SD)	2.8 N (median); 2.9 ± 0.8 N (mean ± SD)	−10% (reported tendency)

Principal comparison: U-test with Bonferroni correction, α = 0.05; material-series comparisons at 1,000-cycle intervals (Table 5).

Qualification: Group F provides eligible ceramic-primary evidence; other groups are within-report metal controls. Table 4 values and reported percentage tendencies retained. The narrative statement that group F retained 75% of its initial force conflicts with Table 4 and is not used. Individual specimens split equally between two testing devices.

S1 ID 41: Nakagawa et al. (2017), Dental Materials Journal [39]

Testing: insertion/removal cycles: No cyclic aging; five pull-offs per load; chewing/mechanical loading: NR; thermocycling: NR.

Conditions: NR; withdrawal/crosshead speed: 40 mm/min; seating: 50 or 100 N for 15 s. Summary measure: Mean; SD NR.

Group / condition S1 ID 41	Primary material	Secondary material	Taper or geometry	Group n / unit	Initial or principal force	Final/post-aging force	Absolute or percentage change
50 N seating; gap 0 µm	Ce-TZP/Al2O3	Ce-TZP/Al2O3	2°	5 secondaries	NR (group-specific means only in graphs)	NR	NR
100 N seating; gap 0 µm	Ce-TZP/Al2O3	Ce-TZP/Al2O3	2°	5 secondaries	NR (group-specific means only in graphs)	NR	NR
50 N seating; gap 10 µm	Ce-TZP/Al2O3	Ce-TZP/Al2O3	2°	5 secondaries	NR (group-specific means only in graphs)	NR	NR
100 N seating; gap 10 µm	Ce-TZP/Al2O3	Ce-TZP/Al2O3	2°	5 secondaries	NR (group-specific means only in graphs)	NR	NR
50 N seating; gap 0 µm	Ce-TZP/Al2O3	Ce-TZP/Al2O3	4°	5 secondaries	NR (group-specific means only in graphs)	NR	NR
100 N seating; gap 0 µm	Ce-TZP/Al2O3	Ce-TZP/Al2O3	4°	5 secondaries	NR (group-specific means only in graphs)	NR	NR
50 N seating; gap 10 µm	Ce-TZP/Al2O3	Ce-TZP/Al2O3	4°	5 secondaries	NR (group-specific means only in graphs)	NR	NR
100 N seating; gap 10 µm	Ce-TZP/Al2O3	Ce-TZP/Al2O3	4°	5 secondaries	NR (group-specific means only in graphs)	NR	NR
50 N seating; gap 0 µm	Ce-TZP/Al2O3	Ce-TZP/Al2O3	6°	5 secondaries	NR (group-specific means only in graphs)	NR	NR
100 N seating; gap 0 µm	Ce-TZP/Al2O3	Ce-TZP/Al2O3	6°	5 secondaries	NR (group-specific means only in graphs)	NR	NR
50 N seating; gap 10 µm	Ce-TZP/Al2O3	Ce-TZP/Al2O3	6°	5 secondaries	NR (group-specific means only in graphs)	NR	NR
100 N seating; gap 10 µm	Ce-TZP/Al2O3	Ce-TZP/Al2O3	6°	5 secondaries	NR (group-specific means only in graphs)	NR	NR
50 N seating; both gaps combined	Ce-TZP/Al2O3	Ce-TZP/Al2O3	2°	10 secondaries	23 N (mean; SD NR)	NR	NR
50 N seating; both gaps combined	Ce-TZP/Al2O3	Ce-TZP/Al2O3	4°	10 secondaries	8 N (mean; SD NR)	NR	NR

Group / condition S1 ID 41	Primary material	Secondary material	Taper or geometry	Group n / unit	Initial or principal force	Final/post-aging force	Absolute or percentage change
50 N seating; both gaps combined	Ce-TZP/Al2O3	Ce-TZP/Al2O3	6°	10 secondaries	No retention reported	NR	NR
100 N seating; both gaps combined	Ce-TZP/Al2O3	Ce-TZP/Al2O3	2°	10 secondaries	47 N (mean; SD NR)	NR	NR
100 N seating; both gaps combined	Ce-TZP/Al2O3	Ce-TZP/Al2O3	4°	10 secondaries	15 N (mean; SD NR)	NR	NR
100 N seating; both gaps combined	Ce-TZP/Al2O3	Ce-TZP/Al2O3	6°	10 secondaries	No retention reported	NR	NR

Principal comparison: Two-way ANOVA: taper significant at both loads ($p < 0.05$); gap setting not significant (Results/Table 2).

Qualification: Taper-level mean forces explicitly reported in text are separate from the six taper/gap groups. Graph-only group values were not estimated. Three primary crowns and 30 secondary crowns; repeated use of a primary limits independence. “No retention” is retained as qualitative wording, not converted to zero. One primary was shared within each taper; five secondary crowns were tested per taper/space combination. Taper-level summaries pool the two space settings (ten secondaries).

S1 ID 42: Nakagawa et al. (2017), Journal of Osaka Dental University [40]

Testing: insertion/removal cycles: No cyclic aging; five measurements per seating load; chewing/mechanical loading: NR; thermocycling: NR.

Conditions: NR; withdrawal/crosshead speed: 40 mm/min; seating: 50 and 100 N for 15 s. Summary measure: Point estimates and 99% confidence interval as reported.

Group / condition S1 ID 42	Primary material	Secondary material	Taper or geometry	Group n / unit	Initial or principal force	Final/post-aging force	Absolute or percentage change
Taper factor estimate	Ce-TZP/Al2O3	Ce-TZP/Al2O3	2°	18-condition L18 array; one crown assembly per condition	35.8 N; 99% CI ± 6.6 N (point estimate)	NR	NR
Taper factor estimate	Ce-TZP/Al2O3	Ce-TZP/Al2O3	4°	18-condition L18 array; one crown assembly per condition	15.9 N; 99% CI ± 6.6 N (point estimate)	NR	NR
Taper factor estimate	Ce-TZP/Al2O3	Ce-TZP/Al2O3	6°	18-condition L18 array; one crown assembly per condition	1.4 N; 99% CI ± 6.6 N (point estimate)	NR	NR

Principal comparison: Taguchi L18 design; significant taper effect ($p < 0.01$); main-effect estimates across the orthogonal array.

Qualification: Eight fabrication/design factors were varied in an L18 array. The three rows are reported factor estimates, not independent groups with raw means or SDs. Eighteen individual-condition retention values were not numerically extractable. Experimental overlap with the other Nakagawa report cannot be ruled out.

S1 ID 43: Nakajima et al. (2019) [41]

Testing: insertion/removal cycles: No cyclic aging; five force measurements; chewing/mechanical loading: NR; thermocycling: NR.

Conditions: Dry or distilled water at 37°C; withdrawal/crosshead speed: 40 mm/min; seating: 25, 50 or 100 N for 5 s. Summary measure: Mean; SD NR.

Group / condition S1 ID 43	Primary material	Secondary material	Taper or geometry	Group n / unit	Initial or principal force	Final/post-aging force	Absolute or percentage change
25 N seating; dry	Ce-TZP/Al2O3	Ce-TZP/Al2O3	2° half angle	5 pairs	9.7 N (mean; SD NR)	NR	NR
25 N seating; water	Ce-TZP/Al2O3	Ce-TZP/Al2O3	2° half angle	5 pairs	12.2 N (mean; SD NR)	NR	NR
50 N seating; dry	Ce-TZP/Al2O3	Ce-TZP/Al2O3	2° half angle	5 pairs	20.1 N (mean; SD NR)	NR	NR
50 N seating; water	Ce-TZP/Al2O3	Ce-TZP/Al2O3	2° half angle	5 pairs	22.6 N (mean; SD NR)	NR	NR
100 N seating; dry	Ce-TZP/Al2O3	Ce-TZP/Al2O3	2° half angle	5 pairs	43.0 N (mean; SD NR)	NR	NR
100 N seating; water	Ce-TZP/Al2O3	Ce-TZP/Al2O3	2° half angle	5 pairs	46.3 N (mean; SD NR)	NR	NR
25 N seating; dry	Ce-TZP/Al2O3	Ce-TZP/Al2O3	4° half angle	5 pairs	4.1 N (mean; SD NR)	NR	NR
25 N seating; water	Ce-TZP/Al2O3	Ce-TZP/Al2O3	4° half angle	5 pairs	4.9 N (mean; SD NR)	NR	NR
50 N seating; dry	Ce-TZP/Al2O3	Ce-TZP/Al2O3	4° half angle	5 pairs	9.4 N (mean; SD NR)	NR	NR
50 N seating; water	Ce-TZP/Al2O3	Ce-TZP/Al2O3	4° half angle	5 pairs	10.5 N (mean; SD NR)	NR	NR
100 N seating; dry	Ce-TZP/Al2O3	Ce-TZP/Al2O3	4° half angle	5 pairs	17.7 N (mean; SD NR)	NR	NR
100 N seating; water	Ce-TZP/Al2O3	Ce-TZP/Al2O3	4° half angle	5 pairs	19.8 N (mean; SD NR)	NR	NR

Principal comparison: Wet versus dry and taper comparisons; water increased the reported means. Exact group SDs were not extracted from figures.

Qualification: Same pairs were measured dry and wet at several seating loads. Water comparisons are testing conditions, not aging changes. Source-specific taper terminology retained. The same five pairs per taper underwent the dry/wet and seating-load measurements.

S1 ID 44: Fujiki et al. (2020) [42]

Testing: insertion/removal cycles: No cyclic aging; three force measurements averaged; chewing/mechanical loading: NR; thermocycling: NR.

Conditions: Dry; 21.0 ± 1.2°C; humidity 43.3 ± 8.0%; withdrawal/crosshead speed: 40 mm/min; seating: 25 or 50 N for 5 s. Summary measure: Mean; SD NR.

Group / condition S1 ID 44	Primary material	Secondary material	Taper or geometry	Group n / unit	Initial or principal force	Final/post-aging force	Absolute or percentage change
25 N; polished/non-polished	Ce-TZP/Al2O3	Ce-TZP/Al2O3	2° half angle	5 pairs per taper	9.7 N (mean; SD NR)	NR	NR
25 N; polished/blasted	Ce-TZP/Al2O3	Ce-TZP/Al2O3	2° half angle	5 pairs per taper	10.9 N (mean; SD NR)	NR	NR
25 N; blasted/blasted	Ce-TZP/Al2O3	Ce-TZP/Al2O3	2° half angle	5 pairs per taper	12.3 N (mean; SD NR)	NR	NR
50 N; polished/non-polished	Ce-TZP/Al2O3	Ce-TZP/Al2O3	2° half angle	5 pairs per taper	20.0 N (mean; SD NR)	NR	NR
50 N; polished/blasted	Ce-TZP/Al2O3	Ce-TZP/Al2O3	2° half angle	5 pairs per taper	22.2 N (mean; SD NR)	NR	NR
50 N; blasted/blasted	Ce-TZP/Al2O3	Ce-TZP/Al2O3	2° half angle	5 pairs per taper	27.1 N (mean; SD NR)	NR	NR
25 N; polished/non-polished	Ce-TZP/Al2O3	Ce-TZP/Al2O3	4° half angle	5 pairs per taper	4.4 N (mean; SD NR)	NR	NR
25 N; polished/blasted	Ce-TZP/Al2O3	Ce-TZP/Al2O3	4° half angle	5 pairs per taper	6.1 N (mean; SD NR)	NR	NR
25 N; blasted/blasted	Ce-TZP/Al2O3	Ce-TZP/Al2O3	4° half angle	5 pairs per taper	9.3 N (mean; SD NR)	NR	NR
50 N; polished/non-polished	Ce-TZP/Al2O3	Ce-TZP/Al2O3	4° half angle	5 pairs per taper	9.9 N (mean; SD NR)	NR	NR
50 N; polished/blasted	Ce-TZP/Al2O3	Ce-TZP/Al2O3	4° half angle	5 pairs per taper	13.6 N (mean; SD NR)	NR	NR
50 N; blasted/blasted	Ce-TZP/Al2O3	Ce-TZP/Al2O3	4° half angle	5 pairs per taper	20.5 N (mean; SD NR)	NR	NR

Principal comparison: One-way ANOVA and Tukey comparisons, $\alpha = 0.05$; significant surface-condition differences reported.

Qualification: Eligible crown-pair experiment uses Ce-TZP/Al2O3. Separate flat-specimen friction experiments include Y-TZP and gold; those coupon results are not charted as crown-retention values. Blasting: 50 µm alumina, 0.3 MPa, approximately 10 mm distance, 13 s/cm²; CAD gap 10 µm; secondary wall 0.4 mm.
